# Uncovering Interactions in the Frequency Domain

**DOI:** 10.1371/journal.pcbi.1000087

**Published:** 2008-05-30

**Authors:** Shuixia Guo, Jianhua Wu, Mingzhou Ding, Jianfeng Feng

**Affiliations:** 1 Department of Mathematics, Hunan Normal University, Changsha, China; 2 Department of Computer Science and Mathematics, Warwick University, Coventry, United Kingdom; 3 Crayton Pruitt Family Department of Biomedical Engineering, University of Florida Gainesville, Gainesville, Florida, United States of America; 4 Centre for Computational System Biology, Fudan University, Shanghai, China; University College London, United Kingdom

## Abstract

Oscillatory activity plays a critical role in regulating biological processes at levels ranging from subcellular, cellular, and network to the whole organism, and often involves a large number of interacting elements. We shed light on this issue by introducing a novel approach called partial Granger causality to reliably reveal interaction patterns in multivariate data with exogenous inputs and latent variables in the frequency domain. The method is extensively tested with toy models, and successfully applied to experimental datasets, including (1) gene microarray data of HeLa cell cycle; (2) in vivo multi-electrode array (MEA) local field potentials (LFPs) recorded from the inferotemporal cortex of a sheep; and (3) in vivo LFPs recorded from distributed sites in the right hemisphere of a macaque monkey.

## Author Summary

When predicting the structure of a network (a gene network, a protein network, a metabolic network or a neuronal network) based upon simultaneously recorded multi-variable temporal data, a major tool is either the Bayesian network or the Granger causality. We focused on the Granger causality, and it has become increasingly important in recent years because of the huge body of temporal data available in, for example, molecular biology (microarray gene data) and physiology (multi-electrode array recordings of multi-neurons). However, all methods of estimating the Granger causality tend to ignore latent variables, which are ubiquitous in experimental data. Here, we have developed a method that can eliminate the influence of latent variables in predicting the network structure. The method is then extended to the frequency domain. The ability of the method to eliminate the influence of latent variables is extensively verified in toy models and then applied to a gene circuit, a neuronal network, and a network of brain areas. Both in the time and frequency domains, our approach can be used to detect a network structure when multi-dimensional temporal data are available.

## Introduction

Recently, as reviewed in [1], many novel approaches in molecular biology have been invented to improve the bulk-scale methods that measure average values for a population of genes or proteins and mask their dynamical activities which are critical for the function of cells [2]–[4]. In neurophysiology, there is a long history of analyzing neural dynamics by recording at the single neuron, neuronal network and brain area levels. Based upon such experimental data, how to explore the network structure of genes, proteins, neurons, etc, is one of the most important issues in Systems Biology. In the literature, there exist two closely related approaches (see for example [5]–[8]): Bayesian modeling and Granger causality analysis. The appealing properties of the Granger causality approach are: (1) the flow of time is explicitly used to define causal relationships; (2) there is a frequency decomposition that reveals the frequency at which two units or variables interact with each other.

In the current paper we concentrate on the Granger causality approach. The concept of Granger causality, originally introduced by Wiener [9] and later formalized by Granger [10], has played an important role in investigating the relationship among stationary time series. Specifically, given two time series, if the variance of the prediction error for the second time series at the present time is reduced by including past measurements from the first time series in the (non)linear regression model, then the first time series can be said to cause the second time series. Geweke's decomposition of a vector autoregressive process [11] led to a set of causality measures which have a spectral representation and make the interpretation more informative and useful [12].

**Figure 1:**
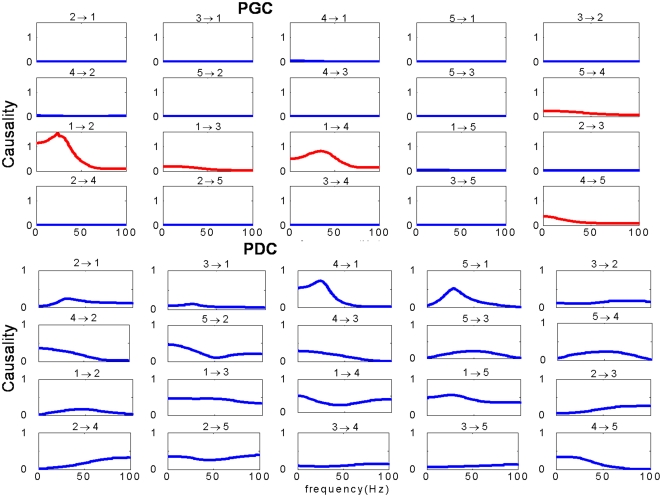
Frequency decomposition. Comparison of the frequency decomposition of all 20 kinds of relationships between the partial Granger causality (PGC) and PDC for data in Example 1 with *a_i_*∼*U*[0,1], *b_i_* = 2, *c_i_* = 5, *i* = 1,…5. Upper panels are the results of PGC, and bottom panels are the results of PDC.

We first develop a novel approach to calculate Granger causality: partial Granger causality, both in the time and the frequency domain, aiming to deal with the case that the data recorded has latent variables. Employing toy models we compare our approach with partial directed coherence (PDC), which is used to detect direct influences in multivariate time series [13]. It is shown that partial Granger causality is able to reveal the right causal relationship whereas PDC fails (see Figure 1). The simple reason is that our decomposition relies on the Kolmogrov equation, but PDC type of approach lacks this property. As a consequence, the results in the frequency domain decomposition could be in conflict with the results in the time domain.

After validating the approach we then apply it to three sets of experimental data. The first data set is microarray data from the IκB-NF-κB circuit in HeLa cell [14]. Although the data has been widely analyzed in the literature, no results in the frequency domain have been reported. Our approach reveals three basic frequencies in the circuit. The first one with a period of 16 hours is the HeLa cycle time. The second one with a period of 4 hours has been reported in the past by other analysis methods [2]–[3]. Finally, the third frequency feature has a period of around 10 hours and has been observed in other gene networks, such as the P53 network [1]. Our causality analysis further reveals how these genes interact with each other at the three identified frequencies in the IκB-NF-κB circuit. The second data set consists of multi-electrode array recordings from the inferotemporal cortex (IT) of a sheep performing a stimulus discrimination task. Five channels are used in our analysis to illustrate the application of our approach. The third set of data is recorded with transcortical bipolar electrodes from 15 distributed sites in the right hemisphere of a monkey trained to perform a visuomotor task. In comparison with the results obtained from the conditional Granger causality analysis, an additional interaction between two areas is found.

## Results

### Toy Model

To illustrate the frequency decomposition of the partial Granger causality introduced here, we first consider a toy model with exogenous inputs and latent variables (see Methods section). In this model 5 simultaneously generated time series are defined by the equations
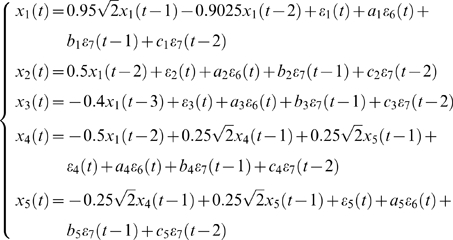
where ε*_i_*(*t*),*i* = 1,2,…7 are zero-mean uncorrelated processes with identical variances, *a_i_*ε_6_is the exogenous input, and the term *b_i_*ε_7_(*t*−1)+*c_i_*ε_7_(*t*−2) represents the influence of latent variables.

**Figure 2:**
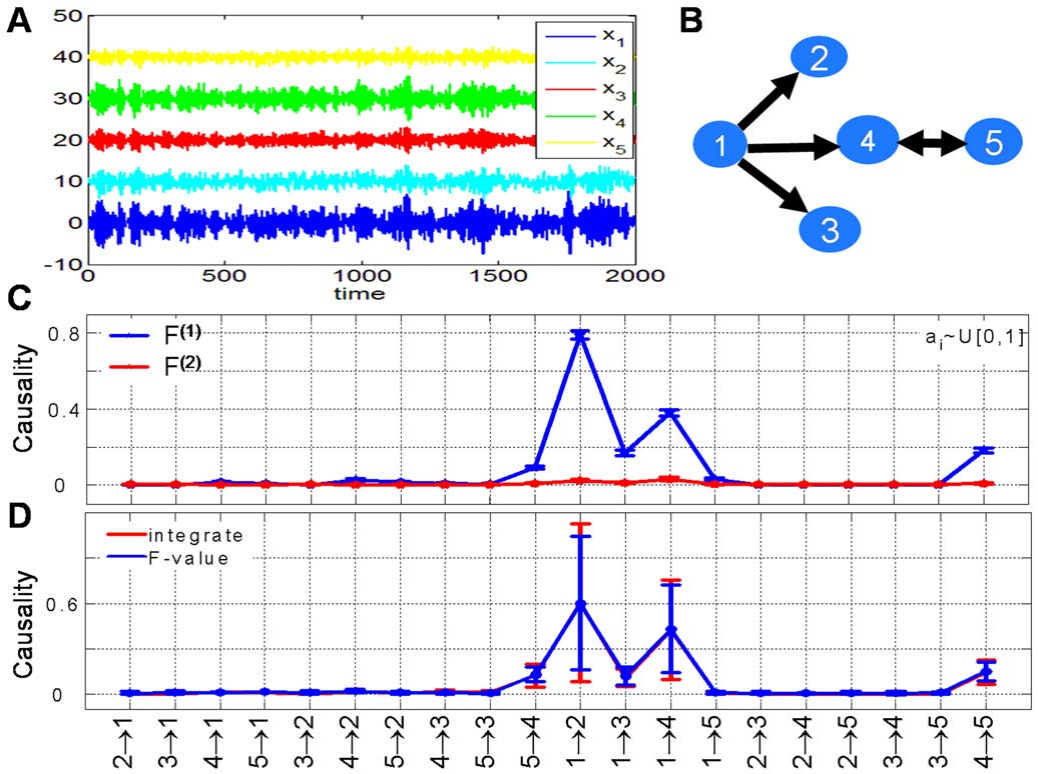
Simulation results. (A) Traces of the time series we considered in Example 1 when *a_i_*∼*U*[0,1], *b_i_* = 2, *c_i_* = 5, *i* = 1,…5. *x*_2_,*x*_3_,*x*_4_ and *x*_5_ are shifted upward for visualization purpose. (B) The causality structure is plotted. (C) Comparison of the partial Granger causality *F* ^(1)^ and the conditional Granger causality *F* ^(2)^ when *a_i_*∼*U*[0,1]. It is obvious that *F* ^(2)^ fails to pick up the correct relationship while the inferred relationship from *F* ^(1)^ is consistent with the true structure (B). (D) Comparison of the partial Granger causality in the time domain and frequency domain when *a_i_*∼*U*[0,1], *i* = 1,…5 in Example 1. The blue line represents the case of time domain, and the red line is the integral of the frequency domain in the interval [−π, π].

From the model, one can see that *x*_1_(*t*) is a direct source to *x*_2_(*t*), *x*_3_(*t*), and *x*_4_(*t*), *x*_4_(*t*) and *x*_5_(*t*) share a feedback loop, and there is no direct connection between *x*_1_(*t*) and *x*_5_(*t*). We perform a simulation of this system with *a_i_*∼*U*[0,1], *b_i_* = 2, *c_i_* = 5, *i* = 1,…5 (we extensively tested our approach in other more general cases of *a_i_*, *b_i_*, *c_i_*, see [13]) to generate a data set of 2000 data points with a sample rate of 200 Hz. Figure 2A illustrates the traces of 5 time series. It is obvious that the system is stationary. The network structure is depicted in Figure 2B. Figure 2C is the comparison between our partial Granger causality *F* ^(1)^ and the conditional Granger causality *F* ^(2)^ [15]. It is clearly shown that our partial Granger causality outperforms the conditional Granger causality. The values of the conditional Granger causality are all very small due to the latent variables and common inputs, while the correct structure is revealed via the partial Granger causality. In particular, the interaction 4→5 is not identified by the conditional causality, but it is correctly revealed in our partial Granger causality approach.

Figure 2D presents a comparison between the time domain partial Granger causality and the frequency domain partial Granger causality. Blue line is the value of the partial Granger causality for all 20 kinds of relationship calculated in the time domain. Red line is the summation (integration) of the partial Granger causality for frequencies in the range of [−π,π]. As expected, Figure 2D demonstrates that the decomposition in the frequency domain fits very well with the partial Granger causality in the time domain.

As mentioned before, PDC has been used in the literature to reveal the causal relationship in the frequency domain [13]. However, it lacks a theoretical foundation. Figure 1 is the detailed comparison of the causality in the frequency domain of all 20 kinds of relationship between the partial Granger causality (PGC) and PDC. The upper panels are the results obtained from the PGC in the frequency domain. It is easy to see that there are direct causal drives from 1 to 2, 3 and 4, and a feedback between 5 and 4. Most importantly it is consistent with the results in the time domain. The bottom panels are the results obtained from PDC. It is evident that the causality for almost all relationship is significant, in contradiction with the results in the time domain. In addition to the exampled considered here, more extensive testing of the PGC has been carried out and comparison made with existing approaches (see Text S1). Next we apply PGC to the experimental data.

### NF-κB: A Tri-Frequency Circuit

We applied partial causality analysis to HeLa cell cycle gene expression data collected by Whitfield et al. (2002) [14]. These data contain three complete cell cycles, i.e., 48 time points distributed at intervals of 1 h, where the HeLa cell cycle is 16 h. This data can be downloaded at http://genome-www.stanford.edu/Human-CellCycle/Hela/. At each time point, there are three or four replicates for each gene selected.

The NF-κB, a stress-regulated transcription factor belonging to the Rel family, plays a pivotal role in the control of inflammatory and innate responses. NF-κB activation has been related to multiple aspects of tumorigenesis, including the control of cell proliferation and migration, cell cycle progression and apoptosis. Whereas only limited information is available regarding the direct involvement of NF-κB in cell-cycle regulation, it was also found that the levels of NF-κB activation are linked to signaling that controls cell-cycle progression in HeLa cells.

**Figure 3:**
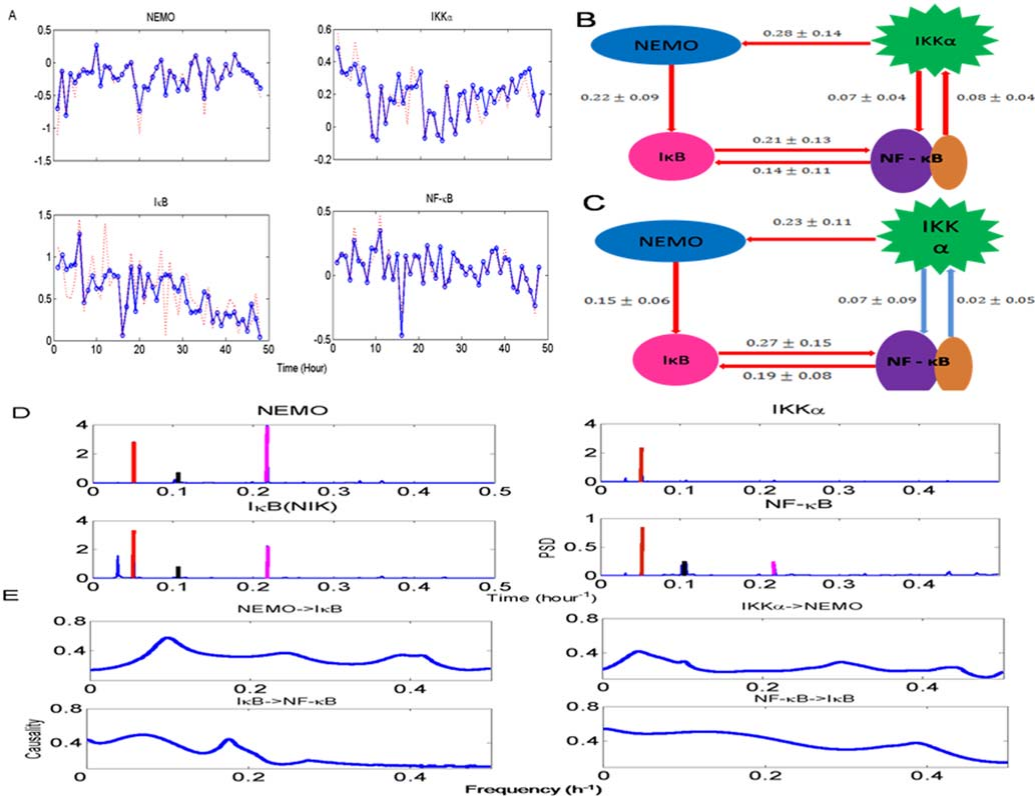
Results for microarray data. (A) Gene expression profile plot of actual data and fitted data by AR model to the NF-κB, NEMO, IKKα, Iand κB genes. A network is composed based on calculated time domain causality for gene module NF-κB, which contains NF-κB, NEMO, IKKα, and IκB genes. (B) is constructed based on pairwise Granger causality method, and (C) is constructed based on PGC method. According to the confidence interval, two connections become insignificant after partial influence is taken into account. (D) Power spectrum density (PSD) for IKKα, NEMO, IκB, and NF-κB genes. There is only one frequency at 16 hours in IKKα, but there are two prominent frequencies for NEMO, IκB, and NF-κB at 16 hours and 4 hours. (E) Frequency domain causality for IKKα, NEMO, IκB, and NF-κB genes.

Here we applied pairwise Granger causality method and partial Granger causality (PGC) methods based on a sliding-window VAR model. We only applied both methods to one typical gene modules, which is regulated by 2 transcription factors, namely: nuclear factor-κB (NF-κB) in the context of cell cycle progression of transformed HeLa cells. In Figure 3A, we plotted the original data and fitted VAR model (dotted lines). The obtained results of Granger causality in the time domain are depicted in Figure 3B (pairwise Granger causality) and in Figure 3C (PGC).

Our gene expression analysis (Figure 3) indicates that the activation of NF-κB correlates with increased activity of IKKα, a natural repressor of IκB -dependent inhibition of NF-κB. As presented in Figure 3B, for the causal network of NF-κB module based on pairwise Granger causality analysis, there are directional connections between IKKα, NEMO and IκB, and also bidirectional connections between IKKα, IκB and NF-κB. Here only the causality that is significant is shown, and the magnitude of the causality and the confidence interval are presented along the arrows. Figure 3C shows the causal network of NF-κB module based on partial Granger causality analysis presented in this paper. Four directional causality connections are preserved and two are eliminated after partial causality analysis is applied. As reported by experimental data, the activity of NF-κB is tightly regulated by its interaction with inhibitory IκB proteins. Activation of NF-κB is achieved through the action of a family of serine/threonine k (IKK). The IKK contains two catalytic subunits (IKKα and IKKβ) and a regulatory/adapter protein NEMO (also known as IKKγ). The causality analysis of NF-κB moduai le presents the activation progression of NF-κB, and it also depicts the causal effect of each gene during the transcription progression. The results indicate that NF-κB transcription factor participates (directly or indirectly) in the control of a complex pattern of HeLa cell cycle regulators in a bidirectional fashion.

As discussed in the Methods section, the Granger causality is consistent in both time and frequency domains. However, it may be more convenient to decompose the time domain causality into its frequency content, such that the profile connections can be examined under a specific frequency. Figure 3D presents the power spectrum plot for four genes. The power of the four genes concentrates on three specific frequencies *f*_1_ = 0.061, *f*_2_ = 0.011, and *f*_3_ = 0.22 h^−1^. Then partial frequency causality in the frequency domain is calculated.

It is interesting to see that the peak of IKKα→NEMO is around 16 hours, which implies that the HeLa cell cycle is originated from the driving of IKKα. The peak causality of NEMO→IκB is at 10 hours. Although the power at 10-hour frequency is less that the other two, it is consistently presented in all genes. To the best of our knowledge, it seems there are no direct reports on the 10-hour frequency of the NF-κB circuit. However, it is reported in, for example, the p53 circuit (see Figure 1A in [1]). The important role played by NF-κB to regulate the p53 circuit has been reported in [16]. The second peak of NEMO→IκB locates at around 4-hour frequency and PSD is very significant in both NEMO and IκB. This frequency is reported in, for example, Figure 2 in [3]. From our analysis, we conclude that the 4-hour frequency is generated from NEMO, but it is absent in IKKα. It would be interesting to test this experimentally and single out its functional meaning. Furthermore, the driving from IκB to NF-κB is mainly at 16-hour frequency and its harmonic (8-hour) frequency. Finally the feedback from NF-κB to IκB is less frequency specific.

### Intra-Network Data: Theta-Frequency Circuit

The experimental data set is the local field potential (LFP) data that was collected from the inferotemporal cortex in left and right hemisphere of the sheep. Multi-electrode array recordings consisted of 64 channels in each hemisphere and individual electrodes were fabricated from tungsten wires (125µ diam.) sharpened to a tip smaller than 1 µ and insulated with epoxylite. The sampling frequency for the LFPs was 2000 Hz. The sheep were trained to perform an operant discrimination task in which different pairs of sheep faces were presented and a correct panel-press response elicited a food reward [17]–[21].

Inferior temporal (IT) cortex is considered to be the highest processing stage along the ventral pathway in the visual system. It is implicated in such higher cognitive functions as categorization and memory formation. fMRI study has reported that ventral temporal regions of primates can be differently activated by different visual stimuli, such as faces, houses and other objects [22]. Recently both spikes and local field potentials have been found to be selective to a variety of stimuli and they are tolerant to retinal position and size [23].

**Figure 4:**
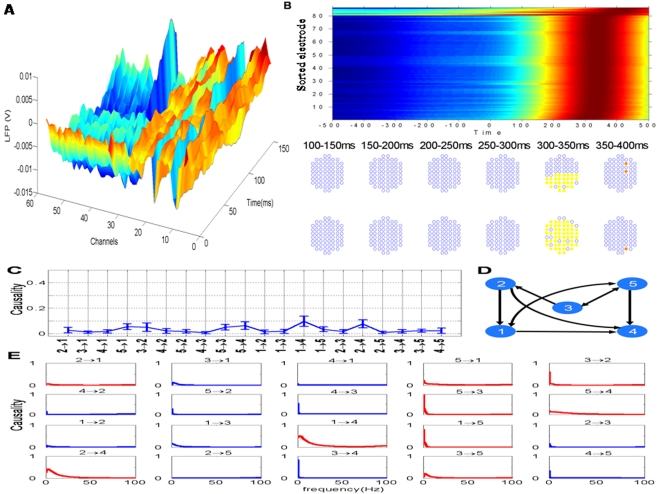
Results for sheep data. (A) Local field potential recorded from 64 channels. Time 0 is the starting point of stimuli. (B) Spatial-temporal pattern of theta power distribution (−500 to 500 ms) across the recording array for one experimental session in response to face presentation. Electrodes are arranged by the latency of the normalized theta power. The electrodes with increased power are marked by the filled colour on the electrode grid in different time slots of 50 ms in the duration 100–400 ms. (C) Partial Granger causality of all possible relationships in time domain. (D) The inferred structure from experimental data in the time domain. (E) The frequency decomposition of all possible relationship is consistent with the structure inferred from time domain.

Much of current studies are based on either neuroimaging or single unit recording techniques. fMRI can accurately locate the brain regions that are active during a visual task but its temporal resolution is poor. Single unit recordings provide direct detailed neuronal information but it is unable to investigate large neuronal ensembles. We use multi-electrode array that consists of up to 128 electrodes and make recordings in sheep IT cortex in both brain hemispheres while animals performed discrimination tasks between pairs of faces and objects (see Figure 4A).

To see the spatial power distribution on the recording array, the electrodes with increased theta power were arranged by the latency of each channel. Figure 4B shows the theta (3–10 Hz) power distribution on the electrode array. The activation of theta power for face stimuli is concentrated within the latency of 300–350 ms. The activated regions include part of the left hemisphere and nearly the whole area of the right hemisphere. The activation region for object presentation is similar in terms of its topographical positions on the array but the stimulus elicited a major activation starting at 200 ms in the left hemisphere which is followed by right hemisphere activation at 250 ms. The sequential activation is also observed for face stimuli in other recording sessions. Assuming that LFP carries input signals from lower brain areas, synchronized theta wave may represent a parallel input into IT while the sequence of theta waves with different latencies may reflect a traveling wave within the recorded region.

It is preferable that all links between distinct pairs of channels (64 channels) be found. However, even with the data size we have at the moment (10 seconds recordings with a sample rate of 2 K Hz), fitting a 64 dimensional model is somewhat problematic. Hence, here we only select five channels to demonstrate the application of our approach and will publish the biological results elsewhere. The links revealed in our approach could be thought of as ‘functional’ interactions between five channels, as defined in the fMRI literature [24]. In fact, the limited data analyzed aligns well with the setup of the current paper: it contains the exogenous input (see below) and latent variables (due to unrecorded inputs and the fact that we only choose five channels).

The partial Granger causality in the time domain is shown in Figure 4C. The complete causal relationship is presented in Figure 4D. In Figure 4E, the partial Granger causality in the frequency domain is depicted. We conclude that the interaction between these channels is in the theta band. For example, the frequency decomposition corresponding to the peak (1→4) in Figure 4C in the time domain has a peak around 10 Hz. Although there are activities in the power spectral density in the gamma band for the five channels (not shown), we have not observed any interactions between these five channels.

### Inter-Network Data: Beta- and Gamma-Frequency Circuit

We refer the reader to [15] for details of the experiment. Briefly, the LFP data were collected when the monkey performed a GO/NO-GO visual pattern discrimination task. The presence of oscillatory field potential activity in the beta (14–30 Hz) frequency range was reported in the sensorimotor cortex during the prestimulus period.

**Figure 5:**
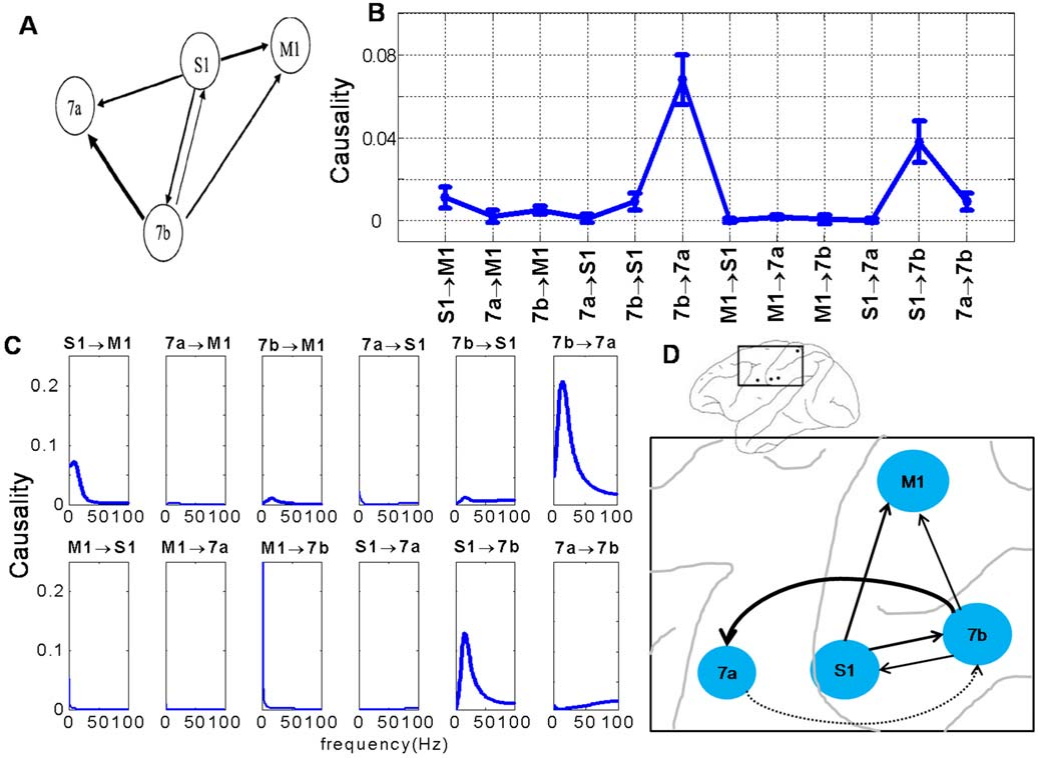
Results for monkey data. (A) Pairwise Granger causality of all possible relationships. (B) The partial Granger causality in the time domain. (C) Frequency decomposition of all possible relationships. (D) The inferred structure from experimental data. The four areas in the brain are marked in the upper trace and the detailed interactions are shown in the bottom trace. The dashed line indicates the additional interaction found using partial Granger causality.

It is pointed out in [15] that if only pairwise Granger causality is applied, the connectivity structure is as depicted in Figure 5A. Using the conditional Granger causality, the causal relationship between the primary somatosensory (S1) and one of the inferior posterior parietal sites (in area 7a) is eliminated, a result predicted from anatomical considerations. Applying the partial Granger causality in the time domain, we obtain the results as shown in Figure 5B, where the actual values and the confidence intervals are depicted. 7b is another inferior posterior parietal site and M1 is the primary motor site. It is clearly seen that there are six causal relationships, i.e., S1→M1, 7b→M1, 7b→S1, 7b→7a, S1→7b, and finally 7a→7b. Figure 5C is the Granger causality in the frequency domain. According to Figure 5B, we see that there are six pairs which have significant Granger causality. The first five have been reported in the literature and are in the Beta band (10–30) Hz. The final one, 7a→7b, has a peak at a high frequency (the super Gamma band). It has been reported in the literature that the nervous systems use different frequency bands to communicate with each other [25].

## Methods

### Partial Granger Causality: Time Domain

Consider a multiple stationary time series of dimension *n*, {*W_t_*}. The series has the following vector autoregressive representation with the use of the lag operator *L*:
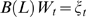
(1)where *E*(ξ*_t_*) = 0, var(ξ*_t_*) = Σ, an *n*×*n* matrix, and *B* is a polynomial matrix of *L*. *B*(0) = *I_n_*, *I_n_* is an *n*×*n* identity matrix. Now suppose that *W_t_* has been decomposed into three vectors (measured variables) *X_t_*, *Y_t_*, and *Z_t_* with *k*, *l*, and *m* dimensions, respectively, i.e., *W_t_* = (*X_t_^T^*,*Y_t_^T^*,*Z_t_^T^*)*^T^*, where (.)*^T^* denotes matrix transposition.

Generally, the perturbation ξ*_t_* in Equation 1 can be represented as a noise term ε*_t_* together with an exogenous term *E_t_^x^* and a latent variable term *L_t_^a^* ^1^. Equation 1 can be rewritten as
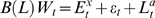
(2)where the random vectors (*E_t_^x^*,*L_t_^a^*) and ε*_t_* are independent. The exogenous variable *E_t_^x^* represents the environmental drive and is typically present in any experimental setup. For example, all neurons in the inferior temporal cortex receive inputs from lower visual areas such as V1 and V2 and the incoming signal could be represented as exogenous variables. The latent variable *L_t_^a^* is a variable that cannot be measured in the experiment.

The vector autoregressive representation for *W* involving three time series *X_t_* (*k* dimensional vector), *Y_t_* (*l* dimensional vector) and *Z_t_* (*m* dimensional vector) can be written in the following way:
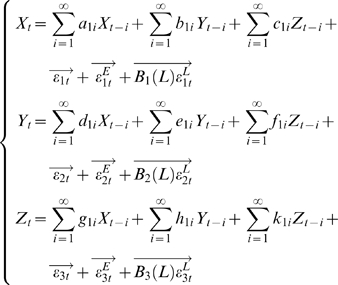
(3)where 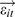,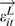,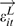 are normally distributed random vectors and *B_i_*(*L*) is a polynomial matrix of *L* of appropriate size.

For simplicity of notation, let us define
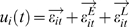

*i* = 1,2,3. The noise covariance matrix for Equation 3 can be represented as
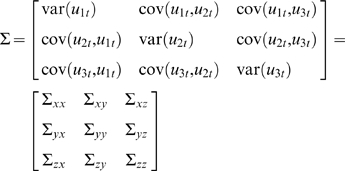
Following the idea of Granger causality, let us further consider two time series *X_t_* and *Z_t_* (to fit *X_t_* and *Z_t_* in *W* exclusively using *X* and *Z*) (due to Wald representation, latent variables can be represented as the summation of normally distributed random inputs, depending on history), the joint autoregressive representation for *X_t_* and *Z_t_* can be written as
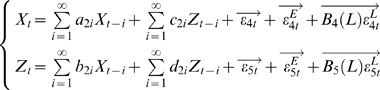
(4)The noise covariance matrix for Equation 4 can be represented as
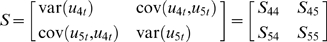
We have defined partial Granger causality in the time domain (see Text S1), which reflects the causal influence from *Y* to *X* conditioned on *Z* by eliminating the influence of common exogenous inputs and latent variables. It has the following expression:
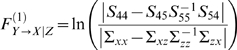
(5)It is interesting to compare *F* ^(1)^ with the definition of the conditional Granger causality *F* ^(2)^ defined by
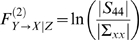
(6)Note that the main difference between the conditional and the partial Granger causality is that in the definition of the conditional Granger causality, the effect of latent and exogenous variables is not eliminated both in the denominator term |Σ*_xx_*| and in the numerator |*S*_44_|. In our definition of the partial Granger causality, we use the conditional variance in both the denominator |Σ*_xx_*−Σ*_xz_*Σ*_zz_* ^−1^Σ*_zx_*| and the numerator |*S*_44_−*S*_45_ *S*_55_^−1^ *S*_54_|. As a result, the effect of the latent and exogenous variables could be eliminated. This was proven to be important as demonstrated extensively in the Results section and in Text S1. Of particular interest is that the definition of the partial Granger causality has a transparent statistical meaning since it depends on a well-understood notation: the conditional variance.

To deal with exogenous inputs and latent variables is one of the central topics in statistics and, as one could expect, there is an extensive literature on the topic. On page 353 in [26], for example, the author has raised the issue and gone further on page 355 to define the partial directed correlation. However, our approach is completely different. First of all, the partial Granger causality is based upon the definition of the conditional Granger causality, which is proved to be one of the most widely used Granger causality definition [27] in the literature. The statistical meaning is transparent, as discussed in the paragraph above. Secondly, as also mentioned above, we extend the time domain partial Granger causality to the frequency domain in the next subsection, which is one of the most appealing properties of the Granger causality.

### Partial Granger Causality: Frequency Domain

To drive the spectral decomposition of the time domain partial Granger causality, we first multiply the matrix
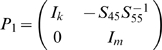
(7)to both sides of Equation 4. The normalized equations are represented as:
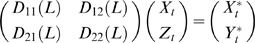
(8)with *D*_11_(0) = *I_k_*, *D*_22_(0) = *I_m_*, *D*_21_(0) = 0, cov(*X_t_* ^*^,*Z_t_* ^*^) = 0. We note that var(*X_t_* ^*^) = *S*_44_−*S*_45_ *S*_55_^−1^ *S*_54_, var(*Z_t_* ^*^) = *S*_55_. For Equation 3, we also multiply
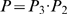
(9)where
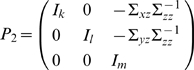
(10)and
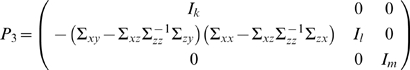
(11)to both sides of Equation 3. The normalized equation of Equation 3 becomes
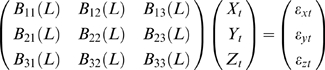
(12)where ε*_xt_*, ε*_yt_*, ε*_zt_* are independent, and their variances being Σˆ*_xx_*, Σˆ*_yy_*, Σˆ*_zz_* with
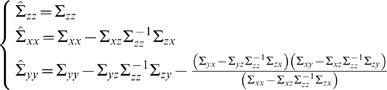
After Fourier transforming Equation 8 and Equation 12, we can rewrite these two equations in the following expression:
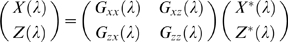
(13)and
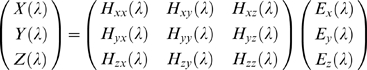
(14)Noting that *X*(λ) and *Z*(λ) from Equation 13 are identical with that from Equation 14, we thus have
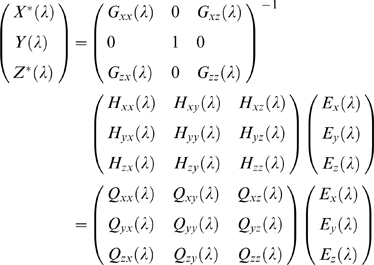
(15)where *Q*(*λ*) = *G* ^−1^(*λ*)*H*(*λ*). Now the power spectrum of *X* ^*^ is
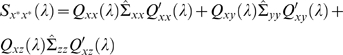
(16)where (.)′ denotes the complex transformation and conjunction. In light of the fact that Σˆ*_xx_* = Σ*_xx_*−Σ*_xz_*Σ*_zz_* ^−1^Σ*_zx_*, the first term in Equation 16 can be thought of as the intrinsic power after eliminating exogenous inputs and latent variables and the remaining two terms as the combined causal influence from *Y* mediated by *Z*. This interpretation leads immediately to the definition
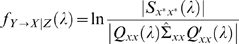
(17)Note that according to Equation 8, the variance of *X* ^*^ equals *S*_44_−*S*_45_ *S*_55_^−1^ *S*_54_. By the Kolmogrov formula [28] for spectral decompositions and under some mild conditions, the Granger causality in the frequency domain and in the time domain measures satisfies
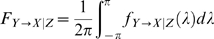
(18)The dependence of partial Granger causality on the coefficient of VAR model is quite complex. Further discussion is presented in Text S2.

## Discussion

We have presented a study on the frequency decomposition for the partial Granger causality. The time domain partial Granger causality and its frequency domain decomposition are successfully applied to toy models and experimental data.

### Partial Granger Causality and Its Frequency Domain Decomposition

In the literature various definitions of the Granger causality in the frequency domain have been introduced. For more than three time series, Kalminski and Blilowska [29] proposed a full multi-variate spectral measure, called directed transfer function (DTF), which is used to determine the directional influences between any given pair of variables in a multivariate data set. Sameshima and Baccala [30] introduced PDC to detect direct influence in multivariate time series. Earlier, Geweke [11] has introduced the conditional Granger causality to infer the original direct relationship between multi-variable time series, as recently reviewed in [15], [27]–[28]. In [11] both a time domain measure, consistent with that of Granger, and its frequency decomposition were given. However, when the exogenous inputs or latent variables are present, the conditional Granger causality fails to identify the correct causal relationship while the partial Granger causality we defined this paper remains robust against the exogenous input and latent variables (see Figure 2), as pointed out in Text S1. One of the key properties of the conditional Granger causality of Geweke's formulation is that the summation of the Granger causality in the frequency domain equals the Granger causality in the time domain. This is due to the Kolmogrov equation for frequency decompositions. Both PDC and DTF lack this property and the inferred structures could simply be misleading. Here we follow the idea of Geweke's formulation and the partial Granger causality in the frequency domain is given.

One of our aims of the current paper is to present a method to correctly calculate the Granger causality when there are latent variables and exogenous inputs. Our results on toy models have demonstrated that the Granger causality defined here is robust against latent variables and exogenous inputs, in comparison with the quantities such as the conditional Granger causality etc. The other aim is to demonstrate that an *ad hoc* definition of the causality such as PDC in the frequency domain could be misleading. It usually yields contradicting results between the time domain Granger causality and the frequency domain Granger causality.

### HeLa Gene Network

Due to the limitation of HeLa microarray data (sampling rate is one hour), we are not able to assess the fast dynamical activity which occurs at a minute scale. It is pointed out in [31] that there are two pathways in NF-κB circuit: one is canonical (fast time scale, minutes) and the other is non-canonical (slow time scale, hours or days). The canonical pathway involves NEMO and is faster than the non-canonical pathway which does not involve NEMO. Our results contradict the above conclusion. The non-canonical pathway does involve NEMO, although it exhibits a slow dynamics. Of course, we do not exclude the possibility that the causality between IKKα and NEMO is due to the crosstalk between canonical and non-canonical pathways. However, one thing is certain. The claim that ‘It is important to note that non-canonical activation of NF-κB appears to lack highly dynamic control’ [31] seems untrue. The NF-κB circuit clearly shows a tri-frequency activity and the causality between each gene is strong or significant.

### Gamma, Beta, and Theta Rhythms

Gamma rhythms occur during persistent, self-sustained activity and are a hallmark of cortical activity during sensory processing and cognition. Beta oscillatory activity is often observed to be synchronized between various parts of sensorimotor cortex. Theta-frequency activity is observed during some short term memory tasks and reflects the on-line state of the hippocampus; one of readiness to process incoming signals [32]. In our data, although theta wave is observable for most channels (see Figure 4B), they are synchronous at around 300 ms, which is more or less the time of the evolved field potential. In conclusion, there are different frequencies in the recorded brain activity and their interactions give rise to different cognitive functions.

## Supporting Information

Text S1 Partial Granger causality: eliminating exogenous inputs and latent variables.
(0.50 MB PDF)

Text S2 A simple example in the frequency domain: dependence of the Granger causality on model parameters.
(0.04 MB PDF)
