# Supplementary material for: Uncovering Interactions in the Frequency Domain
Source: PLoS Comput Biol. 2008 May 30;4(5):e1000087. doi: 10.1371/journal.pcbi.1000087 (PMC2398781; doi:10.1371/journal.pcbi.1000087)
Supplement: Text S1 — Partial Granger causality: eliminating exogenous inputs and latent variables. (0.50 MB PDF) [file pcbi.1000087.s001.pdf]

Supplemental Material I: Partial Granger Causality–Eliminating Exogenous Inputs and Latent Variables

April 9, 2008

Abstract

Attempts to identify causal interactions in multi-variable biological time series (e.g., gene data, protein data, physiological data) can be undermined by the confounding influence of environmental (exogenous) inputs. Compounding this problem, we are commonly only able to record a subset of all related variables in a system. These recorded variables are likely to be influenced by unrecorded (latent) variables. To address this problem, we introduce a novel variant of a widely used statistical measure of causality - Granger causality - that is inspired by the definition of partial correlation. Our 'partial Granger causality' measure is extensively tested with toy models, both linear and nonlinear, and is applied to experimental data: *in vivo* multi-electrode array (MEA) local field potentials (LFPs) recorded from the inferotemporal cortex of sheep. Our results demonstrate that partial Granger causality can reveal the underlying interactions among elements in a network in the presence of exogenous inputs and latent variables in many cases where the existing conditional Granger causality fails.

1 Introduction

Methods for identifying intrinsic relationships among elements in a network are increasingly in demand in today's data-rich research areas such as biology and economics. In particular, advances in experimental and computational techniques are revolutionizing the field of neuroscience. On one hand novel experimental techniques such as high-density multi-electrode arrays (MEAs) have made routine the acquisition of massive amounts of empirical data. On the other, new computational techniques are increasingly in demand for interpreting this data and for generating hypotheses.

A question of great interest with respect to network interactions is whether there exist causal relations among a set of measured variables (Baker et al., 2006; Datta et al., 1997; Fairhall et al., 2006; Feng et al., 2005; Gourevitch et al., 2007; Jacobi et al., 2007; Miller et al., 2007; Oswald et al., 2007; Seth, in press). Over the last few decades several techniques such as Bayesian networks (Friedman et al., 2000) and Granger causality (Baccalá et al., 1998; Gourevitch et al., 2006; Granger, 1969; Granger, 1982) have been developed to identify causal relationships in dynamic systems. Wiener (Wiener, 1965) proposed a way to measure the causal influence of one time series on another by conceiving the notion that the prediction of one time series could be improved by incorporating knowledge of the other. Granger (Granger, 1969) formalized this notion in the context of linear vector autoregression (VAR) model of stochastic processes. Specifically, given two time series, if the variance of the prediction error for the second time series is reduced by including past measurements of the first time series in the linear regression model, then the first time series can be said to cause the second time series. From this definition it is clear that the flow of time plays a vital role in the inference of directed interactions from time series data and as such many applications of Granger causality remain in the time domain. Granger's conception of causality has received a great deal of attention and has been applied widely in the econometrics literature and more recently in the biological literature (Baccalá et al., 1998; Gourevitch et al., 2006; Granger, 1969; Granger, 1982). The formalism for bivariate Granger causality is described in Appendix I (section 6.1).

The basic Granger causality described in Appendix I (6.1) is applicable only to bivariate time series. In multivariable (more than two) situations, one time series can be connected to another time series in a direct or an indirect manner, raising the important question of

whether there exists a (direct) causal influence between two time series when the influence of other time series are taken into account. In this case, repeated bivariate analysis can be misleading. For example, one time series may falsely appear to cause another if they are both influenced by a third time series but with different delays. To address this issue, Geweke introduced conditional Granger causality (Geweke, 1984), as recently reviewed in (Chen et al., 2006; Ding et al., 2006). Conditional Granger causality is based on a straightforward expansion of the autoregressive model to a multivariate case including all measured variables. In this case, one variable causes a second variable if the prediction error variance of the first is reduced after including the second variable in the model, with all other variables included in both cases. The formalism for conditional Granger causality is given in Appendix I (6.2).

Critically, the ability of conditional Granger causality to deal with indirect interactions depends on being able to measure all relevant variables in a system. Often, this is not possible, and both environmental (exogenous) inputs and unmeasured latent variables can confound accurate causal influences. For example, in our experimental data recorded from the inferotemporal (IT) cortex of sheep, every measured neuron receives common exogenous inputs from the visual cortex and feedback from the prefrontal cortex. Moreover, even with advanced MEA techniques, we are only able to record a tiny subset of interacting neurons in a single area and there are bound to be latent variables. *Hence controlling for exogenous inputs and latent variables is a critical issue when applying Granger causality to experimental data.* In this article, inspired by the definition of the partial correlation in statistics, we introduce a novel definition of partial Granger causality to confront exactly this problem.

The paper is organized as follows. First, we introduce the partial Granger causality and discuss in theoretical terms its advantages over conditional Granger causality. Both Granger causalities are then extensively tested in linear, nonlinear models with and without exogenous inputs and latent variables. We find that when the exogenous input is absent or small, both conditional and partial Granger causality can correctly infer the underlying causal relationships. However, when the exogenous input is sufficiently strong enough, or when the data contains both exogenous inputs and latent variables, the conditional Granger causality fails to pick up the correct causal relationship while the partial Granger causality remains robust. Finally, we apply both Granger causality measures to local field potential (LFP) recordings from the inferotemporal cortex of sheep.

2 Partial Granger causality

2.1 Linear partial Granger causality

2.1.1 Model

Both bivariate and conditional Granger causality have been extensively discussed in the neuroscience literature and elsewhere (e.g., Gourevitch et al., 2007). We provide the formalisms for these measures in Appendix I and proceed directly to the formulation of partial Granger causality.

For three time series X_t, Y_t and Z_t, define $\overrightarrow{y_t} = (X_t, Y_t, Z_t)$, where X_t, Y_t and Z_t with k, l and m dimensions, respectively. A general form of an autoregressive model with zero mean and exogenous variable $\overrightarrow{\epsilon_t^E}$ has the following vector autoregressive representation

with the use of the lag operator $\mathcal{L}$:

$$\mathbf{B}(\mathcal{L})\overrightarrow{y}_t = \overrightarrow{\epsilon_t^E} + \overrightarrow{\epsilon_t} \tag{1}$$

where $\mathbf{B}$ is a polynomial matrix of $\mathcal{L}$, $\mathbf{B}(0) = I_n$, the $n \times n$ identity matrix. The two random vectors $\overrightarrow{\epsilon^E}$ and $\overrightarrow{\epsilon}$ are independent. The exogenous variable $\overrightarrow{\epsilon^E}$ represents the environmental drive and is typically present in any experimental setup. For example, all neurons in the inferotemporal cortex receive inputs from visual cortex. Not that each element of the vector $\overrightarrow{\epsilon_t^E}$ could be different.

As already mentioned, the confounding influence of latent variables is possibly even more disruptive than that due to exogenous inputs. To incorporate latent variables, assume that the i'th network element receives unmeasured inputs of the form $\sum_{j=1}^N x_{ij}(t)/N$ (see Fig. 1 (**A**)), where each x_{ij} is a stationary time series and j is the latent index.

According to the Wold representation, any stationary variable $\xi(t)$ can be expressed as the summation of the form $\sum_k \psi_k \epsilon(t-k)$, and we have

$$x_{ij}(t) = \sum_{k=1} \psi_{ij,k} \epsilon_{ij}^L (t-k).$$

Therefore

$$\begin{aligned} \textstyle\sum_{j=1}^N x_{ij}(t)/N &= \textstyle\sum_{j=1}^N \sum_{k=1} \psi_{ij,k} \epsilon_{ij}^L (t-k)/N \\ &= \textstyle\sum_{k=1} \bar{\psi}_{i,k} \epsilon_i^L (t-k) \end{aligned}$$

where $\psi_.$ are constants. In words, each network element receives a latent input which depends on the history So the model Eq. (1) becomes

$$\mathbf{B}(\mathcal{L})\overrightarrow{y_t} = \overrightarrow{\epsilon_t^E} + \overrightarrow{\epsilon_t} + \mathbf{B}^{(1)}(\mathcal{L})\overrightarrow{\epsilon_t^L} \tag{2}$$

where the random vectors $(\overrightarrow{\epsilon_t^E}, \overrightarrow{\epsilon_t^L})$ and $\overrightarrow{\epsilon_t}$ are independent and $\mathbf{B}^{(1)}(\mathcal{L})$ is another polynomial matrix of $\mathcal{L}$ of appropriate size.

To illustrate how to eliminate the influences of exogenous inputs and latent variables let us first consider an example. Suppose we have three independent variables X, Y and Z with variance x^2, y^2 and z^2 respectively. Another three variables ξ, η and ζ are functions of X,Y and Z and are defined by

$$\begin{cases} \xi &= X + Z \\ \eta &= Y + Z \\ \zeta &= Z \end{cases} \tag{3}$$

Here Z represents an exogenous input to ξ and η. If we eliminate the influence of Z, ξ and η should be uncorrelated. The covariance matrix of ξ, η and ζ can be expressed as

$$\Omega = \begin{pmatrix} x^2 + z^2 & z^2 & z^2 \\ z^2 & y^2 + z^2 & z^2 \\ z^2 & z^2 & z^2 \end{pmatrix}.$$

If we consider

$$\text{cov}(\xi, \eta) - \text{cov}(\xi, \zeta)\text{cov}(\zeta, \zeta)^{-1}\text{cov}(\zeta, \eta)$$

we see that the expression above equals zero as one would expect. Actually this is exactly the partial correlation defined in statistics. This simple example above leads us to the following definition of partial Granger causality.

Consider two time series X_t and Z_t which admit a joint autoregressive representation of the form

$$\begin{cases} X_t &= \sum_{i=1}^{\infty} a_{1i} X_{t-i} + \sum_{i=1}^{\infty} c_{1i} Z_{t-i} + \overrightarrow{\epsilon_{1t}} + \overrightarrow{\epsilon_{1t}^E} + \overrightarrow{B_1(\mathcal{L})\epsilon_{1t}^L} \\ Z_t &= \sum_{i=1}^{\infty} b_{1i} Z_{t-i} + \sum_{i=1}^{\infty} d_{1i} X_{t-i} + \overrightarrow{\epsilon_{2t}} + \overrightarrow{\epsilon_{2t}^E} + \overrightarrow{B_2(\mathcal{L})\epsilon_{2t}^L} \end{cases} \tag{4}$$

For simplicity of notation, let us define

$$u_i(t) = \overrightarrow{\epsilon_{it}} + \overrightarrow{\epsilon_{it}^E} + \overrightarrow{B_i(\mathcal{L})\epsilon_{it}^L}$$

$i = 1, 2$. The noise covariance matrix for the model can be represented as

$$S = \begin{bmatrix} \text{var}(u_{1t}) & \text{cov}(u_{1t}, u_{2t}) \\ \text{cov}(u_{2t}, u_{1t}) & \text{var}(u_{2t}) \end{bmatrix}$$

Extending this concept, the vector autoregressive representation for a system involving three variables X_t, Y_t and Z_t can be written as follows:

$$\begin{cases} X_t &= \sum_{i=1}^{\infty} a_{2i} X_{t-i} + \sum_{i=1}^{\infty} b_{2i} Y_{t-i} + \sum_{i=1}^{\infty} c_{2i} Z_{t-i} + \overrightarrow{\epsilon_{3t}} + \overrightarrow{\epsilon_{3t}^{E}} + \overrightarrow{B_3(\mathcal{L})\epsilon_{3t}^{L}} \\ Y_t &= \sum_{i=1}^{\infty} d_{2i} X_{t-i} + \sum_{i=1}^{\infty} e_{2i} Y_{t-i} + \sum_{i=1}^{\infty} f_{2i} Z_{t-i} + \overrightarrow{\epsilon_{4t}} + \overrightarrow{\epsilon_{4t}^{E}} + \overrightarrow{B_4(\mathcal{L})\epsilon_{4t}^{L}} \\ Z_t &= \sum_{i=1}^{\infty} g_{2i} X_{t-i} + \sum_{i=1}^{\infty} h_{2i} Y_{t-i} + \sum_{i=1}^{\infty} k_{2i} Z_{t-i} + \overrightarrow{\epsilon_{5t}} + \overrightarrow{\epsilon_{5t}^{E}} + \overrightarrow{B_5(\mathcal{L})\epsilon_{5t}^{L}} \end{cases} \quad (5)$$

The noise covariance matrix for the model can be represented as

$$\Sigma = \begin{bmatrix} \text{var}(u_{3t}) & \text{cov}(u_{3t}, u_{4t}) & \text{cov}(u_{3t}, u_{5t}) \\ \text{cov}(u_{4t}, u_{3t}) & \text{var}(u_{4t}) & \text{cov}(u_{4t}, u_{5t}) \\ \text{cov}(u_{5t}, u_{3t}) & \text{cov}(u_{5t}, u_{4t}) & \text{var}(u_{5t}) \end{bmatrix}$$

where

$$u_i(t) = \overrightarrow{\epsilon_{it}} + \overrightarrow{\epsilon_{it}^{E}} + \overrightarrow{B_i(\mathcal{L})\epsilon_{it}^{L}}$$

$i = 3, 4, 5$.

In order to consider the influence from Y to X controlling for the effect of the exogenous input, we partition the noise covariance matrix S in the following way

$$S = \left[\begin{array}{c|c} \text{var}(u_{1t}) & \text{cov}(u_{1t}, u_{2t}) \\ \hline \text{cov}(u_{2t}, u_{1t}) & \text{var}(u_{2t}) \end{array}\right] = \left[\begin{array}{c|c} S_{11} & S_{12} \\ \hline S_{21} & S_{22} \end{array}\right] \quad (6)$$

Hence the variance of u_{1t} by eliminating the influence of u_{2t} is given by

$$\begin{aligned} R_{XX|Z}^{(1)} &= \text{cov}(u_{1t}, u_{1t}) - \text{cov}(u_{1t}, u_{2t})\text{cov}(u_{2t}, u_{2t})^{-1}\text{cov}(u_{2t}, u_{1t}) \\ &= S_{11} - S_{12} S_{22}^{-1} S_{21} \end{aligned}$$

For the matrix Σ, by eliminating the second row and the second column, we can partition the remaining noise covariance matrix Σ_1 in the following way

$$\Sigma_1 = \left[\begin{array}{c|c} \text{var}(u_{3t}) & \text{cov}(u_{3t}, u_{5t}) \\ \hline \text{cov}(u_{5t}, u_{3t}) & \text{var}(u_{5t}) \end{array}\right] = \left[\begin{array}{c|c} \Sigma_{11} & \Sigma_{12} \\ \hline \Sigma_{21} & \Sigma_{22} \end{array}\right] \tag{7}$$

We can define the variance of u_{3t} by eliminating the influence of u_{5t} similarly.

$$\begin{aligned} R^{(2)}_{XX|Z} &= \text{cov}(u_{3t}, u_{3t}) - \text{cov}(u_{3t}, u_{5t})\text{cov}(u_{5t}, u_{5t})^{-1}\text{cov}(u_{5t}, u_{3t}) \\ &= \Sigma_{11} - \Sigma_{12}\Sigma_{22}^{-1}\Sigma_{21} \end{aligned}$$

The value of $R^{(1)}_{XX|Z}$ measures the accuracy of the autoregressive prediction of X based on its previous values conditioned on Z by eliminating the influence of the common exogenous input and latent variables, whereas the value of $R^{(2)}_{XX|Z}$ represents the accuracy of predicting present value of X based on the previous history of both X and Y conditioned on Z by eliminating the influence of the exogenous input and latent variables. According to the causality definition of Granger, if the prediction of one process is improved by incorporating the information of the second process, then the second process causes the first process. Similarly we can define this causal influence by

$$F_1 = \ln\left(\frac{|R^{(1)}_{XX|Z}|}{|R^{(2)}_{XX|Z}|}\right) = \ln\left(\frac{|S_{11} - S_{12}S_{22}^{-1}S_{21}|}{|\Sigma_{11} - \Sigma_{12}\Sigma_{22}^{-1}\Sigma_{21}|}\right) \tag{8}$$

We call F_1 *partial Granger causality.*. For comparison, the standard conditional Granger causality F_2 is defined by

$$F_2 = \ln\left(\frac{|S_{44}|}{|\Sigma_{xx}|}\right). \tag{9}$$

Note that the analogy between partial correlation and partial Granger causality is not exact. Calculation of partial correlation requires knowing the variance of the common input

cov(ζ, ζ) (see Eq. (3)). However, this variance can only be known precisely if the common input can be measured, which is not the case for exogenous inputs or latent variables. The equivalent terms for partial Granger causality, cov(u_{2t}, u_{2t}) and cov(u_{5t}, u_{5t}) reflect elements of both the unmeasured and measured variables.

In theory, we are only able to deal the model with common exogenous inputs and latent variables (the coefficients are all identical). Otherwise, due to Wold representation, any intrinsic variable such as X_t could be expressed as the summation of ϵ_{t-k} and treated as an exogenous and a latent variable as well. However, in our numerical examples below, we try with the model with different coefficients to test the robustness of our approach. As clearly shown in the numerical experiments (Section 3) partial Granger causality performs substantially better than the standard conditional Granger causality in most cases.

A theoretical discussion of the relationship between F_1 and F_2 is given in Appendix II. The essential difference is that conditional Granger causality, the effect of latent and exogenous variables remains present both in the denominator term $|\Sigma_{xx}|$ and in the numerator term $|S_{44}|$. By contrast, in our definition of partial Granger causality, we use the conditional variance in both the denominator $|\Sigma_{xx} - \Sigma_{xz}\Sigma_{zz}^{-1}\Sigma_{zx}|$ and numerator $|S_{44} - S_{45}S_{55}^{-1}S_{54}|$. As a result, the effects of the latent and exogenous variables are taken into account. Of particular interest is that the definition of the partial Granger causality has a transparent statistical meaning since it depends on the well understood notation: the conditional variance.

2.2 Nonlinear partial Granger causality

Our discussion so far has been based on linear VAR models, however neural systems are known to be nonlinear. Recently, several attempts to extend the linear causality to nonlinear causality have been proposed. In (Chen et al., 2004) local linear methods in reduced neighborhoods are considered and the average causality index, over the whole data set, is proposed as a nonlinear measure. In (Ancona et al.,2004) a radial basis function (RBF) approach was used to model data. In (Marinazzo et al., 2006) a nonlinear parametric model for Granger causality of time series was proposed. In this section, we use the method proposed in (Marinazzo et al., 2006) and extend the linear partial Granger causality to nonlinear partial Granger causality.

Let $\mathbf{X}(t)$, $\mathbf{Y}(t)$ and $\mathbf{Z}(t)$ be three time series of k, l, m simultaneously measured quantities, we assume that the time series are stationary. We use the same notation as in (Marinazzo et al., 2006) and aim at quantifying how significant is the effect of Y on X when conditioned on Z. Let us now consider the general nonlinear model

$$\begin{cases} \mathbf{X}(t) &= \sum_j [\mathbf{w}_{1j} \cdot \Phi_j(\mathbf{X}(t-j)) + \mathbf{w}_{1j} \cdot \Phi_j(\mathbf{Z}(t-j))] + u_1 \\ \mathbf{Z}(t) &= \sum_j [\mathbf{w}_{2j} \cdot \Phi_j(\mathbf{X}(t-j)) + \mathbf{w}_{2j} \cdot \Phi_j(\mathbf{Z}(t-j))] + u_2 \end{cases} \tag{10}$$

where u_i represents the error term including exogenous inputs and latent variables and w_{ij} are the coefficients to be fitted. As in (Marinazzo et al., 2006), Φ_j can be selected as the kernel function of $\mathbf{X}$ and $\mathbf{Z}$ and have the following expression:

$$\Phi_j(\mathbf{X}) = \exp(-||\mathbf{X} - \bar{X}_j||^2 / 2\sigma_X^2)$$

$$\Phi_j(\mathbf{Z}) = \exp(-||\mathbf{Z} - \bar{Z}_j||^2 / 2\sigma_Z^2)$$

where $\bar{X}_j, \bar{Z}_j$ are centers of X and Z, $\sigma^2_{X_j}, \sigma^2_{Z_j}$ are variance of X and Z. The parameters $\{\mathbf{w}\}$ must be fixed to minimize the prediction error. The covariance matrix of prediction error in eq. (10) can be expressed as

$$S = \left[\begin{array}{c|c} \mathrm{var}(u_{1t}) & \mathrm{cov}(u_{1t}, u_{2t}) \\ \hline \mathrm{cov}(u_{2t}, u_{1t}) & \mathrm{var}(u_{2t}) \end{array}\right] = \left[\begin{array}{c|c} S_{11} & S_{12} \\ \hline S_{21} & S_{22} \end{array}\right]$$

A joint autoregressive representation has the following expression:

$$\begin{cases} \mathbf{X}(t) &= \sum_j [\mathbf{w}_{3j} \cdot \Phi_j(\mathbf{X}(t-j)) + \mathbf{w}_{3j} \cdot \Phi_j(\mathbf{Y}(t-j)) + \mathbf{w}_{3j} \cdot \Phi_j(\mathbf{Z}(t-j))] + u_3 \\ \mathbf{Y}(t) &= \sum_j [\mathbf{w}_{4j} \cdot \Phi_j(\mathbf{X}(t-j)) + \mathbf{w}_{4j} \cdot \Phi_j(\mathbf{Y}(t-j)) + \mathbf{w}_{4j} \cdot \Phi_j(\mathbf{Z}(t-j))] + u_4 \\ \mathbf{Z}(t) &= \sum_j [\mathbf{w}_{5j} \cdot \Phi_j(\mathbf{X}(t-j)) + \mathbf{w}_{5j} \cdot \Phi_j(\mathbf{Y}(t-j)) + \mathbf{w}_{5j} \cdot \Phi_j(\mathbf{Z}(t-j))] + u_5 \end{cases} \quad (11)$$

Where Φ_j is the kernel function of $\mathbf{Y}$. The covariance matrix of prediction error of the first and the third equations in eq. (11) can be expressed as

$$\Sigma_1 = \left[\begin{array}{c|c} \mathrm{var}(u_{3t}) & \mathrm{cov}(u_{3t}, u_{5t}) \\ \hline \mathrm{cov}(u_{5t}, u_{3t}) & \mathrm{var}(u_{5t}) \end{array}\right] = \left[\begin{array}{c|c} \Sigma_{11} & \Sigma_{12} \\ \hline \Sigma_{21} & \Sigma_{22} \end{array}\right]$$

Similarly, we can define

$$F_1 = \ln\left(\frac{|S_{11} - S_{12}S_{22}^{-1}S_{21}|}{|\Sigma_{11} - \Sigma_{12}\Sigma_{22}^{-1}\Sigma_{21}|}\right)$$

as the nonlinear partial Granger causality which has the same properties as linear partial Granger causality.

As in the case of linear VAR modelling, to fit but not over-fit the data with a nonlinear VAR kernel model is a challenging problem. Not surprisingly, there exists a large literature devoted to model order selection. Example methods include information criteria such as the Akaike criterion (AIC), or dividing the data set into training and generalization data sets (the so-called kennel methods). In our numerical experiments we use the AIC method (see section 3.2); for further discussion see (Bishop 1998).

3 Numerical examples

3.1 Linear model with various exogenous inputs and latent variables

To illustrate and compare the difference between conditional Granger causality (Appendix I) and the partial Granger causality introduced here, we first consider toy models with various exogenous inputs and latent variables. The first toy model we use has been extensively applied (e.g., Ding et al.,2006; Baccalá et al., 2001) in tests of Granger causality. Here we modify this model by adding a common exogenous input and latent variable to each time series. In all figures below, a causal connection illustrated as part of the network if and only if the lower bound of the 95% confidence interval of the (partial, conditional) Granger causality is greater than zero.

Example 1 Suppose that 5 simultaneously generated time series were generated by the equations:

$$
\begin{cases}
x_1(t) &= 0.95\sqrt{2}x_1(t-1) - 0.9025x_1(t-2) + \epsilon_1(t) + a_1\epsilon_6(t) \\
& \quad +b_1\epsilon_7(t-1) + c_1\epsilon_7(t-2) \\
x_2(t) &= 0.5x_1(t-2) + \epsilon_2(t) + a_2\epsilon_6(t) + b_2\epsilon_7(t-1) + c_2\epsilon_7(t-2) \\
x_3(t) &= -0.4x_1(t-3) + \epsilon_3(t) + a_3\epsilon_6(t) + b_3\epsilon_7(t-1) + c_3\epsilon_7(t-2) \\
x_4(t) &= -0.5x_1(t-2) + 0.25\sqrt{2}x_4(t-1) + 0.25\sqrt{2}x_5(t-1) + \epsilon_4(t) \\
& \quad +a_4\epsilon_6(t) + b_4\epsilon_7(t-1) + c_4\epsilon_7(t-2) \\
x_5(t) &= -0.25\sqrt{2}x_4(t-1) + 0.25\sqrt{2}x_5(t-1) + \epsilon_5(t) + a_5\epsilon_6(t) \\
& \quad +b_5\epsilon_7(t-1) + c_5\epsilon_7(t-2)
\end{cases}
$$

where $\epsilon_i(t), i = 1, 2, \cdots, 7$ are zero-mean uncorrelated process, and their variances are

$0.8, 0.6, 1, 1.2, 1, 0.9, 1$ respectively. $a_i, b_i, c_i, i = 1, \cdots, 5$ are parameters, $a_i\epsilon_6$ is then the exogenous input. $b_i\epsilon_7(t-1) + c_i\epsilon_7(t-2)$ are treated as the latent variables to each entity. As discussed in Eq. (2), the coefficients a_i, b_i, c_i depend on i.

Inspection of the above equations reveals that $x_1(t)$ is a direct source to $x_2(t), x_3(t)$, that $x_4(t)$, $x_4(t)$ and $x_5(t)$ share a feedback loop and that there is no direct connection between $x_1(t)$ and $x_5(t)$. Fig. 1 (B) (left) is an example of the 2000 time-steps of the data. The network structure is depicted in Fig. 1 (B) (right). A direct check of the companion matrix tells us that the time series is stationary.

Fig. 1 around here

Firstly, we analyze a simple case. We set $b_i = 0, c_i = 0, i = 1, \cdots, 5$ which means no latent variables are present. We also suppose that all parameters $a_i, i = 1, \cdots, 5$ are identical (later we will relax this assumption). In order to analyze the impact of the common exogenous input on the causality, we compute the partial Granger causality F_1 and conditional Granger causality F_2 in three conditions with a_i equal to 0, 1 and 5 respectively. When $a_i = 0, i = 1, \cdots, 5$, the common exogenous input is absent. The greater the value of a_i, the greater is the influence of the common exogenous input on the time series. F_1 and F_2 for all edges are calculated.

We use the bootstrap approach to construct confidence intervals. Specifically, we simulate the fitted VAR model to generate a data set of 2000 realizations of 2000 time points. In Fig. 2, the histogram of F_1 and F_2 is plotted when $a_i = 0, b_i = 0, c_i = 0, i = 1, \cdots, 5$. It is interesting to see that F_2 is always nonnegative, but F_1 can be negative due to the additional term in its definition. In all our examples, we use 3σ as the confidence interval.

In Fig. 3, the causality values calculated in terms of F_1 and F_2 are shown for different

parameters $a_i, i = 1, \cdots, 5$. The left panels show the magnitudes and confidence intervals of all edges of F_1 and F_2. Blue solid lines represent the value of F_1 and red dotted lines represent the value of F_2. The right panels are the inferred structures of the network corresponding to different a_i. The results show that when $a_i, i = 1, \cdots, 5$ are small (see **(A)**), both conditional Granger causality and partial Granger causality can correctly infer the network structure. However, when $a_i, i = 1, \cdots, 5$ are sufficiently large (see **(B)**), the magnitude of F_2 is very small, falsely implying the absence of any inter-element relationship, which is consistent with the preceding theoretical analysis.

Fig. 2 around here

Fig. 3 around here

As mentioned before, it is almost always the case that in a complex biological network only a small subset of interacting variables will be measured. These variables will be influenced by many latent variables and the confounding influence of latent variables can be more disruptive than that due to exogenous input. In the previous examples, the number of links inferred according to F_2 is always less than F_1: no false positive interactions are introduced by using F_2. With the introduction of latent variables, we will see that this is not always the case.

In example 1, we assume $a_i \sim U[0, 1], i = 1, \cdots, 5$ are random variables with the uniform distribution in $[0, 1]$ and set b_i and c_i to different values (but equal for all i). In this case, the exogenous inputs have in general different magnitudes for different time series, and latent variables are present. The results presented in Fig. 4 clearly demonstrate that F_1 is superior to F_2 in revealing the underlying network interactions. It is clear that when the influence of latent variables is significant, F_1 remain robust while F_2 is unable to identify

the correct underlying relationships.

Fig. 4 around here

In Fig. 5, we relax the assumption that $b_i, i = 1, \cdots, 5$ are the same. We now allow them to impose different influences for different time series and we recalculate F_1 and F_2 and their confidence interval. The figure shows that even in this case, the correct causal relationship is recovered when using F_1. However, the causal relationships inferred by F_2 now include several false positives and is substantially incorrect. These numerical results further confirm that partial Granger causality performs better than conditional Granger causality in the presence of exogenous inputs and latent variables. The results are also consistent with the theoretical explanation of the relationship between F_1 and F_2 as presented in Appendix II.

Fig. 5 around here

3.2 Nonlinear model with various exogenous inputs and latent variables

Next we turn our attention to nonlinear models.

Example 2: We modify the model in example 1 to a series of nonlinear equations as

follows:

$$\begin{cases} x_1(t) &=& 0.125\sqrt{2}\exp\left(-\frac{x_1(t-1)^2}{2}\right) + \epsilon_1(t) + a_1\epsilon_6(t) + b\epsilon_7(t-1) + b\epsilon_7(t-2) \\ x_2(t) &=& 1.2\exp\left(-\frac{x_1(t-1)^2}{2}\right) + \epsilon_2(t) + a_2\epsilon_6(t) + b\epsilon_7(t-1) + b\epsilon_7(t-2) \\ x_3(t) &=& -1.05\exp\left(-\frac{x_1(t-1)^2}{2}\right) + \epsilon_3(t) + a_3\epsilon_6(t) + b\epsilon_7(t-1) + b\epsilon_7(t-2) \\ x_4(t) &=& -1.15\exp\left(-\frac{x_1(t-1)^2}{2}\right) + 0.2\sqrt{2}\exp\left(-\frac{x_4(t-1)^2}{2}\right) \\ & & +1.35\exp\left(-\frac{x_5(t-1)^2}{2}\right) + \epsilon_4(t) + a_4\epsilon_6(t) + b\epsilon_7(t-1) + b\epsilon_7(t-2) \\ x_5(t) &=& -0.5\sqrt{2}\exp\left(-\frac{x_4(t-1)^2}{2}\right) + 0.25\sqrt{2}\exp\left(-\frac{x_5(t-1)^2}{2}\right) \\ & & +\epsilon_5(t) + a_5\epsilon_6(t) + b\epsilon_7(t-1) + b\epsilon_7(t-2) \end{cases}$$

Fig. 6 around here

We use these equations to generate time series of length 2000. An example of the generated time series is shown in Fig. 6 (**A**). The underlying causal relationships are the same as in Example 1. The mean and variance of each time series is chosen as the center and variance in the kernel function (see Section 2.2). The Granger causality values of F_1 and F_2 and corresponding causal network structures are shown in Fig. 6 (**B**) and (**C**). It is clear that when there are exogenous inputs and latent variables in the nonlinear time series, the nonlinear partial Granger causality can reveal the correct underlying relationship while the conditional Granger causality fails to do so, as in the linear case.

Note that we chose the equations in Example 2 to generate nonlinear dynamics of a form well suited to the exponential functions used as kernels in the general nonlinear model (equation 9). Importantly, the general model is not limited to this case and can fit arbitrary nonlinear dynamics given appropriate methods for kernel selection (Marinazzo et al., 2006). Here we omit this step because our aim is only to demonstrate that the par-

tial Granger causality does not depend on linear modelling, and is not to discuss nonlinear autoregressive modelling *per se*.

4 Application to example experimental data

Our final example application is to LFP data collected from sheep inferotemporal cortex. This data was collected using 64-channel MEAs implanted into in each cortical hemisphere. Individual electrodes were fabricated from tungsten wires (125μ diam.) sharpened to a $<$ 1μ tip and insulated with epoxylite. LFPs were sampled at 2000 Hz for 10 seconds per trial. The sheep were trained to perform an operant discrimination task in which different pairs of sheep faces were presented and a correct panel-press response elicited a food reward. Previous analyses of this data can be found elsewhere (Horton et al., 2006; Wu et al., 2007).

Numerical limitations prevent fitting 64-variable multivariate regression models given our data. Therefore, we select only five channels by way of illustration. Clearly, our chosen data exemplifies the issues confronted by our theoretical approach. The data is influenced both by substantial exogenous input (see below) and by a large set of latent variables (due to unrecorded inputs and the fact that we only choose five channels). We note that the links revealed in our approach can be considered 'functional' interactions between five channels, as in the fMRI literature (Kim et al., 2007).

For the selected five channels $(x_1(t), x_2(t), \cdots, x_5(t))$, we have the following correla-

tion matrix:

$$\begin{pmatrix} 1 & 0.9980 & 0.0702 & 0.1696 & 0.0678 \\ 0.9980 & 1 & 0.0713 & 0.1693 & 0.0603 \\ 0.0702 & 0.0713 & 1 & 0.9545 & 0.2751 \\ 0.1696 & 0.1693 & 0.9545 & 1 & 0.2828 \\ 0.0678 & 0.0603 & 0.2751 & 0.2828 & 1 \end{pmatrix} \qquad (12)$$

In order to compare F_1 and F_2 we generate additional data sets in which a common input $\sigma\epsilon(t)$ is added to each channel, i.e. the signals are $y_1(t) = x_1(t) + \sigma\epsilon(t), \cdots, y_5(t) = x_5(t) + \sigma\epsilon(t)$. For example, when the exogenous input variance is $\sigma = 0.01$, the correlation matrix becomes

$$\begin{pmatrix} 1 & 0.9999 & 0.9225 & 0.9338 & 0.9657 \\ 0.9999 & 1 & 0.9231 & 0.9453 & 0.9396 \\ 0.9225 & 0.9231 & 1 & 0.9939 & 0.9323 \\ 0.9338 & 0.9453 & 0.9939 & 1 & 0.9293 \\ 0.9657 & 0.9396 & 0.9323 & 0.9293 & 1 \end{pmatrix} \qquad (13)$$

We then estimate both partial Granger causality and conditional Granger causality for these matrices for different $\sigma = 0.01$. Because the underlying data generating process is not known, we must also estimate the optimal model order (number of time-lags to include in the model). Following standard practice, the optimal model order p is determined according to the Akaike Information Criterion (AIC): $\text{AIC}(p) = N\log(\det(\Sigma)) + 2L^2p$, where Σ is the estimated prediction error covariance matrix with a p-th order model, N is the length of data points and $L (L = 5)$ is the number of variables. The optimal model order corresponds to the minimum AIC which in this case is $p = 10$. The following results use this model

order. To verify robustness we have also used $p = 10, 11, \cdots, 20$, the inferred causal relationships are the same in all cases.

With $\sigma = 0$ (i.e., without any artificially induced exogenous input), Fig. 7(**A**) shows that F_1 and F_2 identify different network structures. Apparently, F_2 suggests additional causal connections such as $2 \rightarrow 3$ and $5 \rightarrow 1$. Based on the numerical analyses above, these additional links are suggestive of the influence of latent variables (Example 1 in the previous section, see Fig. 5). Also, some identified by F_1 are not identified by F_2; for example $2 \rightarrow 5$ and $5 \rightarrow 2$. This is suggestive of the influence of exogenous inputs (Example 1 in the previous section).

Although our theoretical analysis gives reason to believe that partial Granger causality is likely to be more accurate than conditional Granger causality in inferring causal relationships, this is difficult to establish with certainty in this case because we do not know the underlying data generating process (the neural mechanism). However, the two measures can be further compared by their performance when σ is changed, i.e., when artificial exogeneity is introduced. If partial Granger causality is more accurate than conditional Granger causality we would expect it to be robust to these disruptions.

Fig. 7(**B,C** shows the causal network structures inferred by F_1 and F_2 when σ is increased to 0.005 and to 0.01. The structure obtained by F_1 is always robust, but it changes with σ for F_2: when $\sigma = 0.005$, latent links ($2 \rightarrow 3$ and $5 \rightarrow 1$) still exist, see Fig. 7(**B**), but some links such as $3 \rightarrow 4$ and $5 \rightarrow 4$ are broken. When $\sigma = 0.01$, Fig. 7 (**C**) the only connections identified by F_2 are not identified by F_1 $2 \rightarrow 3$ and $5 \rightarrow 1$), suggesting the influence of latent inputs. In general, the obtained results are similar to the examples in the previous section: F_1 is robust, but F_2 is not when there are exogenous inputs and latent

variables.

Fig. 7 around here

5 Discussion

The myriad network structures that comprise the central nervous system are among the most complex objects in nature. Moreover, even with limited experimental access we commonly face the problem of having too much data rather than not enough. The rapid development of novel experimental methods will further exacerbate this problem. New statistical and computational methods are therefore required in order to make sense of this data and by doing so to generate an improved understanding of central nervous system structure and function.

Many neurophysiological methods involve simultaneous recording of time series data from many elements. A key question in this context is how to reveal reliably the interactions among these elements? Over the last decade, Granger causality has become established as a useful method for addressing this question. Applications of Granger causality to physiological data such as spike trains, local field potential, fMRI BOLD signals, and the like, have been very successful and increasingly common. However, as we emphasized, previous applications of Granger causality to biological data may have generated misleading causal network structures due to the influence of exogenous inputs and latent variables. In this paper we have confronted this important problem by introducing a new definition of Granger causality, partial Granger causality, which is robust against various perturbations due to these common inputs.

Our partial Granger causality is inspired by the definition of partial correlation but the analogy is not exact. Partial correlation is able to fully eliminate the influence of additional variables on the measured correlation, but only because the variances of these additional variables are known. Partial Granger causality faces the problem that these variances are not known and must be estimated from combinations of the measured variables (if these variances could be measured directly then standard conditional Granger causality could be used). For this reason, partial Granger causality cannot eliminate the influence of exogenous inputs and latent variables in all cases. Full elimination is only possible if all common inputs have equal influence on all measured variables. However, our theoretical analysis and numerical results together show that in a wide variety of cases, partial Granger causality significantly outperforms conditional Granger causality. Importantly these include cases in which exogenous inputs (Figure 4) and latent inputs (Figure 5) have differing influences on the measured variables. These findings support the notion that partial Granger causality is of substantial practical value for attempts to identify causal networks from time series data.

In this paper we have only considered Granger causality in time domains. Geweke (Geweke, 1982) expanded on Granger's definition by providing a spectral decomposition of the VAR process (Geweke, 1982). This decomposition leads to a set of causality measures which have a frequency-dependent representation and are therefore of particular relevance to neurobiological data (Wu et al., 2007). Future work will explore a spectral version of the present partial Granger measure. In the time domain, after fitting the data with a VAR model, whether there is a causal relationship between X and Y could be statistically assessed via their coefficients. Of course, the advantage of Granger causality over the

simple approach mentioned above is that we also have a quantitative measurement.

Finally, another popular approach for inferring causality among network elements is the Bayesian approach. Another difference between the Bayesian approach and the Granger approach is that Bayesian approaches are not able easily to incorporate feedback interactions, which is certainly limiting in the case of physiological data (see for example Pearl, 1998).

Acknowledgements We benefited from discussions with R. Krishna. Dr. S. Kalvala carefully read the manuscript. J.F. was partially supported by grants from UK EPSRC (EP/C51338X), (EP/D051916), (GR/S30443) and BBSRC (BB/E005802).

References

Akaike, H. Fitting Autoregressive Models for Regression. Annals of the Institute of Statistical Mathematics, 1969;21: 243-7.

Ancona N, Marinazzo D, Stramaglia S. Radial Basis Function Approach to Nonlinear Granger Causality of Time Series. Phys. Rev. E, 2004;70:056221.

Baccalá L, Sameshima K, Ballester G, Valle AC, Timo-Iaria C. Studying the Interaction between Brain Structures via Directed Coherence and Granger Causality. Appl. Signal Process, 1998; 5: 40-48.

Baccalá L, Sameshima K. Partial Directed Coherence: a New Conceptin Neural Structure Determination. Biological Cybernetics, 2001;84: 463-74.

Baker SN, Chiu M, and Fetz EE. Afferent Encoding of Central Oscillations in the Monkey Arm . J Neurophysiol, 2006;95: 3904 - 10.

Bishop CM *Neural Networks and Machine Learning*.1998; Springer: Berlin. Chen Y, Bressler SL, Ding M. Frequency Decomposition of Conditional Granger Causality and Application to Multivariate Neural Field Potential Data. J Neurosci Methods, 2006;150: 228-37.

Chen Y, Rangarajan G, Feng J, Ding M. Analyzing Multiple Nonlinear Time Series with Extended Ganger Causality. Physics Lett. A, 2004;324:26-35.

Datta S, Siwek DF. Excitation of the Brain Stem Pedunculopontine Tegmentum Cholinergic Cells Induces Wakefulness and REM Sleep. J Neurophysiol, 1997;77: 2975 - 88.

Ding M, Chen Y, Bressler SL. Granger Causality: Basic Thoery and Application to Neuroscience. Handbook of Time Series Analysis, ed. Schelter,B., Winterhalder,M.,Timmer,J. Wiley-VCH Verlage, 2006: 451-74.

Fairhall AL, Burlingame CA, Narasimhan R, Harris RA, Puchalla JL, and Berry, II MJ. Selectivity for Multiple Stimulus Features in Retinal Ganglion Cells. J Neurophysiol, 2006;96: 2724 - 38.

Feng J, Durand MD. Decrease in Synaptic Transmission Can Reverse the Propagation Direction of Epileptiform Activity in Hippocampus In Vivo. J Neurophysiol, 2005;93: 1158 - 64.

Friedman N, Linial M, Nachman I, Peer D. Using Bayesian Networks to Analyze Expression Data, RECOMB 00: Proceedings of the Fourth Annual International Conference on Computational Molecular Biology, 2000: 127-35.

Geweke J. Measurement of Linear Dependence and Feedback Between Multiple Time Series. Journal of the American Statistical Association, 1982;77: 304-13.

Geweke J. Measures of Conditional Linear Dependence and Feedback Between Time Series. Journal of the American Statistical Association, 1984;79: 907-15.

Gourevitch B, Eggermont JJ. Evaluating Information Transfer Between Auditory Cortical Neurons. J Neurophysiol, 2007;97: 2533 - 43.

Gourevitch B, Bouquin-Jeannes RL, Faucon G. Linear and Nonlinear Causality Between Signals: Methods, Examples and Neurophysiological Applications. Biol Cybern 2006;95: 349-69.

Gourierous C, Monfort A. Time Series and Dynamic Models, cambridge University Press, London,1997.

Granger C. Investigating Causal Relations by Econometric Models and Cross- spectral Methods. Econometrica, 1969;37: 424-38.

Granger C. Testing for Causality: A Personal Viewpoint. Journal of Economic Dynamics and Control, 1980;2: 329-52.

Horton PM, Nicol AU, Kendrick KM, and Feng J.F. Spike sorting based upon machine learning algorithms (SOMA), J. Neurosci Methods, 2006;160: 52-68.

Knyazeva MG, Fornari E, Meuli R, and Maeder P. Interhemispheric Integration at Different Spatial Scales: The Evidence From EEG Coherence and fMRI. J Neurophysiol, 2006;96: 259 - 75.

Kim J, Zhu W, Chang L. Unified Structural Equation Modeling Approach for the Analysis of Multisubject, Multivariate Functional MRI Data. Human Brain Mapping, 2007; 28: 85-93.

Jacobi S, Moses E. Variability and Corresponding AmplitudeCVelocity Relation of Activity Propagating in One-Dimensional Neural Cultures. J Neurophysiol2007;97:

3597 - 606.

Lutkepohl,H. Introduction to Multiple Time Series Analysis, Springer-Verlag,1993.

Marinazzo D, Pellicoroa M. Nonlinear Parametric Model for Granger Causality of Time Series. Physical Review E, 2006;73:066216.

Miller J, White SA., HVC Neural Sleep Activity Increases with Development and Parallels Nightly Changes in Song Behavior. J Neurophysiol,2007; doi:10.1152/jn.00443.2007

Oswald AM, Doiron B, and Maler L. Interval Coding. I. Burst Interspike Intervals as Indicators of Stimulus Intensity. J Neurophysiol,2007;97: 2731 - 43.

Pearl J. *Causality: Models, Reasoning, and Inference*. 1998;Cambridge University Press: Cambridge, UK.

Seth, A.K. Causal networks in simulated neural systems. Cognitive Neurodynamics. (in press).

Wiener N. The Theory of Prediction. Ed. Beckenbach,E. F., Modern Mathermatics for Engineers, Chap 8. McGraw-Hill, New York,1956.

Wu JH, Kendrick K, Feng J. Detecting Correlation Changes in Electrophysiological Data. Journal of Neuroscience Methods, 2007;161: 155-65.

Wu JH, Liu XG, Feng J. Detecting M:N causality in simultaneously recorded data Journal of Neuroscience Methods, 2007. (accepted).

6 Appendix I: Granger causality

6.1 Pairwise Granger causality

Consider two time series X_t and Y_t. Assume that they are jointly stationary. Individually, under fairly general conditions, each process admits an autoregressive representation

$$\begin{cases} X_t &= \sum_{i=1}^{\infty} a_{1i} X_{t-i} + \epsilon_{1t} \\ Y_t &= \sum_{i=1}^{\infty} b_{1i} Y_{t-i} + \epsilon_{2t} \end{cases} \tag{14}$$

A joint autoregressive representation having information of past measurements of both processes X_t and Y_t can be written as

$$\begin{cases} X_t &= \sum_{i=1}^{\infty} a_{2i} X_{t-i} + \sum_{i=1}^{\infty} c_{2i} Y_{t-i} + \epsilon_{3t} \\ Y_t &= \sum_{i=1}^{\infty} b_{2i} Y_{t-i} + \sum_{i=1}^{\infty} d_{2i} X_{t-i} + \epsilon_{4t} \end{cases} \tag{15}$$

where $\epsilon_{it}, i = 1, 2, 3, 4$ are the prediction error, which are uncorrelated over time. The value of var(ϵ_{1t}) measures the accuracy of the autoregressive prediction of X based on its previous values, whereas the value of var(ϵ_{3t}) represents the accuracy of predicting present value of X based on previous values of both X and Y. According to the causality definition of Granger, if the prediction of one process is improved by incorporating past information of the second process, then the second process causes the first process. In other words, if the variance of prediction error for the first process is reduced by the inclusion of past histories of the second process then a causal relation from the second process to the first process exists. We quantify this causal influence by

$$F_{Y \to X} = \ln(\text{var}(\epsilon_{1t})/\text{var}(\epsilon_{3t})) \tag{16}$$

It is clear that $F_{Y\to X} = 0$ when there is no causal influence from Y to X and $F_{Y\to X} > 0$ when there is. Similarly, one can define causal influence from X to Y as

$$F_{X\to Y} = \ln(\text{var}(\epsilon_{2t})/\text{var}(\epsilon_{4t})) \tag{17}$$

If $F_{X\to Y} = 0$ then there is no causal influence from X to Y otherwise $F_{X\to Y} > 0$ when there is.

6.2 Conditional Granger causality

In real situations, we often face data from multi (more than two) variable time series. A simple approach to such situations is to decompose the multivariable dataset into a series of pairwise analyses, treating each as in the previous section. This approach has some inherent limitations, as amply discussed in the literature (Ding et al., 2006). For example, a pairwise analysis would indicate a causal influence from a process that receives an early input to a process that receives a late input.

To deal properly with the general multivariable case, Geweke (Geweke, 1984) introduced condition Granger causality. This method has the ability to resolve whether the interaction between two time series is direct or is mediated by another recorded time series and whether the causal influence is simply due to different time delays in their respective driving input. Consider two time series X_t and Z_t. The joint autoregressive representation for X_t and Z_t can be written as

$$\begin{cases} X_t &= \sum_{i=1}^{\infty} a_{1i} X_{t-i} + \sum_{i=1}^{\infty} c_{1i} Z_{t-i} + \epsilon_{1t} \\ Z_t &= \sum_{i=1}^{\infty} b_{1i} Z_{t-i} + \sum_{i=1}^{\infty} d_{1i} X_{t-i} + \epsilon_{2t} \end{cases} \tag{18}$$

The noise covariance matrix for the system can be represented as

$$S = \begin{bmatrix} \text{var}(\epsilon_{1t}) & \text{cov}(\epsilon_{1t}, \epsilon_{2t}) \\ \text{cov}(\epsilon_{2t}, \epsilon_{1t}) & \text{var}(\epsilon_{2t}) \end{bmatrix}$$

where var and cov represent variance and co-variance respectively. Extending this representation, the vector autoregressive representation for a system involving three time series X_t, Y_t and Z_t can be written in the following way.

$$\begin{cases} X_t &= \sum_{i=1}^{\infty} a_{2i} X_{t-i} + \sum_{i=1}^{\infty} b_{2i} Y_{t-i} + \sum_{i=1}^{\infty} c_{2i} Z_{t-i} + \epsilon_{3t} \\ Y_t &= \sum_{i=1}^{\infty} d_{2i} X_{t-i} + \sum_{i=1}^{\infty} e_{2i} Y_{t-i} + \sum_{i=1}^{\infty} f_{2i} Z_{t-i} + \epsilon_{4t} \\ Z_t &= \sum_{i=1}^{\infty} g_{2i} X_{t-i} + \sum_{i=1}^{\infty} h_{2i} Y_{t-i} + \sum_{i=1}^{\infty} k_{2i} Z_{t-i} + \epsilon_{5t} \end{cases} \tag{19}$$

The noise covariance matrix for the above system can be represented as

$$\Sigma = \begin{bmatrix} \text{var}(\epsilon_{3t}) & \text{cov}(\epsilon_{3t}, \epsilon_{4t}) & \text{cov}(\epsilon_{3t}, \epsilon_{5t}) \\ \text{cov}(\epsilon_{4t}, \epsilon_{3t}) & \text{var}(\epsilon_{4t}) & \text{cov}(\epsilon_{4t}, \epsilon_{5t}) \\ \text{cov}(\epsilon_{5t}, \epsilon_{3t}) & \text{cov}(\epsilon_{5t}, \epsilon_{4t}) & \text{var}(\epsilon_{5t}) \end{bmatrix}$$

where $\epsilon_{it}, i = 1, \cdots, 5$ are the prediction error, which are uncorrelated over time. From these two sets of equations we define the conditional Granger causality from Y to X conditioned on Z to be

$$F_2 = \ln(|\text{var}(\epsilon_{1t})|/|\text{var}(\epsilon_{3t})|) \tag{20}$$

When the causal influence from Y to X is entirely mediated by Z, the coefficient b_{2i} in eq. (19) is uniformly zero, and var(ϵ_{1t}) = var(ϵ_{3t}). Thus, we have $F_2 = 0$, meaning that no further improvement in the prediction of X can be expected by including past measurements of Y conditioned Z. On the other hand, when there is still a direct influence from Y to X, the inclusion of past measurements of Y in addition to that of X and Z results in better predictions of X, leading to var$(\epsilon_{1t}) >$ var(ϵ_{3t}), and $F_2 > 0$.

7 Appendix II: Relationship between F_1 and F_2 and their confidence intervals

Recall that F_1 represents partial Granger causality and F_2 represents conditional Granger causality. We have

1. F_1 is the extension of F_2, i.e. when there are no common inputs or latent variables, we have $F_1 = F_2$.

2. If Y_t is independent of X_t conditioned on Z_t, then

   - $F_2 = 0$

   - $F_1 \leq 0$. Furthermore, if Y_t is also independent of Z_t, then $F_1 = 0$, otherwise $F_1 < 0$.

3. When $a_i, i = 3, 4, 5$ are sufficiently large and there are no latent variables we have $F_2 \to 0$.

Proof: When there is no common noise and latent variables, by eq. (8) conclusion 1 follows. If Y_t is independent of X_t conditioned on Z_t, then in Eq. (5), all the coefficients $b_{2i} = 0, S_{11} = \Sigma_{11}$, it is clear that $F_2 = 0$.

From Eq. (4) and Eq. (5), we get $u_1(t) = u_3(t), u_{2t} = u_{5t} + \sum\limits_{i=1}^{\infty} h_{2i}^* y_{t-i}$, then

$$
\begin{aligned}
& S_{11} - S_{12} S_{22}^{-1} S_{21} \\
= \;& \text{var}(u_{3t}) - \text{cov}(u_{3t}, u_{5t} + \sum_{i=1}^{\infty} h_{2i}^* y_{t-i})[\text{var}(u_{5t} + \sum_{i=1}^{\infty} h_{2i}^* y_{t-k})]^{-1} \\
& \cdot \text{cov}(u_{5t} + \sum_{i=1}^{\infty} h_{2i}^* y_{t-i}, u_{3t}) \\
= \;& \text{var}(u_{3t}) - \text{cov}(u_{3t}, u_{5t})[\text{var}(u_{5t} + \sum_{i=1}^{\infty} h_{2i}^* y_{t-i})]^{-1} \cdot \text{cov}(u_{5t}, u_{3t}) \\
= \;& \Sigma_{11} - \Sigma_{12} \Sigma_{22}^{-1} \Sigma_{21} - \text{cov}(u_{3t}, u_{5t})[\text{var}(\sum_{i=1}^{\infty} h_{2i}^* y_{t-i})]^{-1} \text{cov}(u_{5t}, u_{3t})
\end{aligned}
$$

If Y_t is also independent of Z_t, then all of the coefficients $h_{2i}^* = 0$, $F_1 = 0$. If Y_t is dependent of Z_t, $S_{11} - S_{12} S_{22}^{-1} S_{21} < \Sigma_{11} - \Sigma_{12} \Sigma_{22}^{-1} \Sigma_{21}$, then $F_1 < 0$. Hence conclusion 2 is true.

The eq. (5) can be expressed in the matrix form as $\mathbf{X}(t) = [X_t, Y_t, Z_t]^T, U(t) = [u_{3t}, u_{4t}, u_{5t}]^T$, and

$$
A(i) = \begin{bmatrix} a_{2i} & b_{2i} & c_{2i} \\ d_{2i} & e_{2i} & f_{2i} \\ g_{2i} & h_{2i} & k_{2i} \end{bmatrix}
$$

then

$$
\mathbf{X}(t) = \sum_{i=1}^{\infty} A(i) \mathbf{X}(t - i) + U(t)
$$

We have

$$
\begin{aligned}
& \text{cov}(\mathbf{X}(t_1), \mathbf{X}(t_2)) \\
= & \text{cov}(\sum_{k_1=1}^{\infty} A(k_1)\mathbf{X}(t_1-k_1) + U(t_1), \sum_{j_1=1}^{\infty} A(j_1)\mathbf{X}(t_2-j_1) + U(t_2)) \\
= & \text{cov}(\sum_{k_1=1}^{\infty} A(k_1)\mathbf{X}(t_1-k_1), \sum_{j_1=1}^{\infty} A(j_1)\mathbf{X}(t_2-j_1)) + \text{cov}(U(t_1), U(t_2)) \\
= & \sum_{k_1=1}^{\infty}\sum_{j_1=1}^{\infty} A(k_1)\text{cov}(\mathbf{X}(t_1-k_1), \mathbf{X}(t_2-j_1))A(j_1)^T + \text{cov}(U(t_1), U(t_2)) \\
= & \sum_{k_1,j_1} A(k_1)(\sum_{k_2,j_2} A(k_2)\text{cov}(\mathbf{X}(t_1-k_1-k_2), \mathbf{X}(t_2-j_1-j_2))A(j_2)^T \\
& +\text{cov}(U(t_1-k_1), U(t_2-j_1)))A(j_1)^T + \text{cov}(U(t_1), U(t_2)) \\
= & \sum_{k_1,k_2,j_1,j_2} A(k_1)A(k_2)\ \text{cov}(\mathbf{X}(t_1-k_1-k_2), \mathbf{X}(t_2-j_1-j_2))A(j_2)^T A(j_1)^T \\
& + \sum_{k_1,j_1} A(k_1)\text{cov}(U(t_1-k_1), U(t_2-j_1))A(j_1)^T + \text{cov}(U(t_1), U(t_2)) \\
= & \cdots \\
= & \sum A(k_1)A(k_2)\cdots A(k_n)\ \text{cov}(\mathbf{X}(t_1-k_1-\cdots-k_n), \mathbf{X}(t_2-j_1-\cdots-j_n)) \\
& \cdot A(j_n)^T A(j_{n-1})^T \cdots A(j_1)^T + \sum A(k_1)A(k_2)\cdots A(k_{n-1}) \\
& \cdot\text{cov}(U(t_1-k_1-\cdots-k_{n-1}), U(t_2-j_1-\cdots-j_{n-1})) \cdot A(j_{n-1})^T A(j_{n-2})^T \cdots A(j_1)^T \\
& + \cdots + \text{cov}(U(t_1), U(t_2))
\end{aligned} \tag{21}
$$

Because ϵ_{it} is uncorrelated over time, u_{it} is also uncorrelated over time. When $a_i, i = 3, 4, 5$ are big and no latent variables, we have

$$
\text{cov}(U(t_1), U(t_2)) = \begin{cases} \sigma^2 \cdot \mathbf{M}, & \text{if } t_1 = t_2 \\ 0, & \text{otherwise.} \end{cases}
$$

where

$$
\mathbf{M} = \sigma^2 \begin{pmatrix} 1+a_3^2 & 1+a_3a_4 & 1+a_3a_5 \\ 1+a_3a_4 & 1+a_4^2 & 1+a_4a_5 \\ 1+a_3a_5 & 1+a_4a_5 & 1+a_5^2 \end{pmatrix}
$$

Denote $u_{1t} = u_{3t} + \sum_{i=1}^{\infty} b_{2i}^* Y_{t-i}$, then

$$\begin{aligned} \text{var}(u_{1t}) &= \text{var}(u_{3t} + \sum_{i=1}^{\infty} b_{2i}^* Y_{t-i}) \\ &= \text{var}(u_{3t}) + \text{var}(\sum_{i=1}^{\infty} b_{2i}^* Y_{t-i}) \\ &= \sigma^2(1 + a_3^2) + \sum_i \sum_j b_{2i}^* b_{2j}^* \text{cov}(Y_{t-i}, Y_{t-j}) \end{aligned}$$

Because $\text{cov}(Y_{t-i}, Y_{t-j})$ is the second row and second column of the above matrix $\text{cov}(\mathbf{X}(t-k), \mathbf{X}(t-j))$, it is clear that $\sigma^2(1 + a_3^2)$ is the only remaining term in Eq. (21), so we have

$$F_2 = \ln \frac{|S_{11}|}{|\Sigma_{11}|} \to 0$$

According to Geweke's formation of Granger causality (Geweke, 1982), the causality between two time series X and Y is decomposed into three terms: two terms are the causality between X and Y, the third term is the instantaneously causality which is the exogenous term in our formulation. The conclusion 3 above tells us the influence of the instantaneously causality on the causality between X and Y. The fact that $F_2 \to 0$ when a_i is small enough is observed in numerical examples.

To estimate the confidence intervals of F_1, we next work on the distribution of F_1. For F_2, we refer the reader to the literature, for example (Geweke, 1982). In our autoregression model (4) and (5), the number of unknown parameters is countably infinite. For the purpose of estimation, here we suppose that all lag lengths have been truncated at p. We further suppose the disturbances in model (4) and (5) are independent and identically distributed, the common distribution is Gaussian.

Let $\mathbf{U}_t = \begin{pmatrix} u_{1t} \\ u_{2t} \end{pmatrix} \sim N(0, S)$, $\mathbf{V}_t = \begin{pmatrix} u_{3t} \\ u_{5t} \end{pmatrix} \sim N(0, \Sigma_1)$, where S and Σ_1 are partitioned as in Eqs. (6) and (7). Then we have the following conclusion.

$$\exp(F_1) \sim F(n-2p-1, n-3p-1). \tag{22}$$

proof: Denote $B = \begin{pmatrix} 1 & -S_{12}S_{22}^{-1} \\ 0 & 1 \end{pmatrix}$, $D = \begin{pmatrix} 1 & -\Sigma_{12}\Sigma_{22}^{-1} \\ 0 & 1 \end{pmatrix}$. $\mathbf{U}^* = B\mathbf{U}, \mathbf{V}^* = D\mathbf{V}$, then we have

$$\mathbf{U}_t^* = \begin{pmatrix} u_{1t}^* \\ u_{2t}^* \end{pmatrix} = \begin{pmatrix} 1 & -S_{12}S_{22}^{-1} \\ 0 & 1 \end{pmatrix} \begin{pmatrix} u_{1t} \\ u_{2t} \end{pmatrix} = \begin{pmatrix} u_{1t} - S_{12}S_{22}^{-1}u_{2t} \\ u_{2t} \end{pmatrix},$$

$$\mathbf{V}_t^* = \begin{pmatrix} u_{3t}^* \\ u_{5t}^* \end{pmatrix} = \begin{pmatrix} 1 & -\Sigma_{12}\Sigma_{22}^{-1} \\ 0 & 1 \end{pmatrix} \begin{pmatrix} u_{3t} \\ u_{5t} \end{pmatrix} = \begin{pmatrix} u_{3t} - \Sigma_{12}\Sigma_{22}^{-1}u_{5t} \\ u_{5t} \end{pmatrix},$$

It is easy to see that

$$\begin{aligned} \text{var}(u_{1t}^*) &= (1, -S_{12}S_{22}^{-1}) \begin{pmatrix} S_{11} & S_{12} \\ S_{21} & S_{22} \end{pmatrix} \begin{pmatrix} 1 \\ -S_{12}S_{22}^{-1} \end{pmatrix} \\ &= S_{11} - S_{12}S_{22}^{-1}S_{21} = R_{XX|Z}^{(1)} \\ \text{var}(u_{3t}^*) &= (1, -\Sigma_{12}\Sigma_{22}^{-1}) \begin{pmatrix} \Sigma_{11} & \Sigma_{12} \\ \Sigma_{21} & \Sigma_{22} \end{pmatrix} \begin{pmatrix} 1 \\ -\Sigma_{12}\Sigma_{22}^{-1} \end{pmatrix} \\ &= \Sigma_{11} - \Sigma_{12}\Sigma_{22}^{-1}\Sigma_{21} = R_{XX|Z}^{(2)} \end{aligned}$$

If model (4) is multiplied by matrix B, then in the new model X_t is a linear function of $X_{t-i}, i = 1, 2, \cdots, p$, $Z_{t-i}, i = 0, 1, \cdots, p$ and a disturbance $u_{1t}^* = u_{1t} - S_{12}S_{22}^{-1}u_{2t}$. Since

the disturbance is uncorrelated with u_{2t}, it is uncorrelated with Z_t, X_{t-i} and $Z_{t-i}, i = 1, 2, \cdots, p$. Similarly, if the first and the third equation of model (5) are multiplied by matrix D, then in the new model X_t is a linear function of $X_{t-i}, Y_{t-i}, i = 1, 2, \cdots, p$, $Z_{t-i}, i = 0, 1, \cdots, p$ and a disturbance $u_{3t}^* = u_{3t} - \Sigma_{12}\Sigma_{22}^{-1}u_{5t}$.

Because $S_{11} - S_{12}S_{22}^{-1}S_{21}$ is the variance of u_{1t}^*, $\Sigma_{11} - \Sigma_{12}\Sigma_{22}^{-1}\Sigma_{21}$ is the variance of u_{3t}^*, we can estimate them by its unbiased variance of prediction error

$$\hat{R}_{XX|Z}^{(1)} = \frac{1}{n-2p-1}\sum_{i=1}^{n}(\hat{u}_{1t}^*)^2$$

$$\hat{R}_{XX|Z}^{(2)} = \frac{1}{n-3p-1}\sum_{i=1}^{n}(\hat{u}_{3t}^*)^2$$

Under the assumption that all the disturbance in Eq. (4) and (5) have identical distribution, u_{1t}^* shares the identical distribution with u_{3t}^* . Then

$$\exp(F_1) = \frac{R_{XX|Z}^{(1)}}{R_{XX|Z}^{(2)}} = \frac{n-3p-1}{n-2p-1} \cdot \frac{\sum\limits_{i=1}^{n}(u_{1t}^*)^2}{\sum\limits_{i=1}^{n}(u_{3t}^*)^2} \sim F(n-2p-1, n-3p-1)$$

This completes the proof.

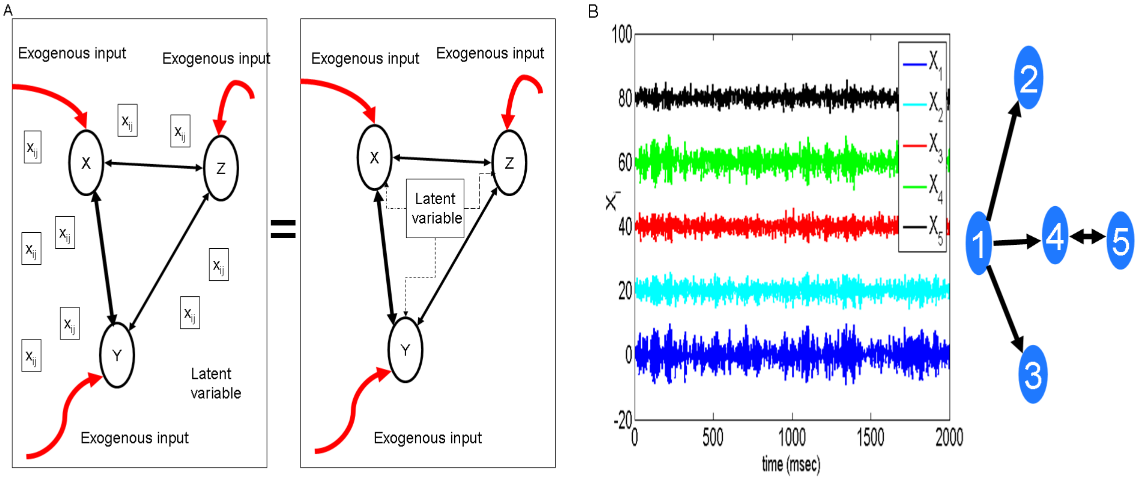

Figure 1: (**A**). A schematic of the network structure of the model considered in the numerical examples. (**B**). Traces of the time series considered in Example 1 (left). x_2, x_3, x_4 and x_5 are shifted upward for visualization purpose. The underlying causal relationships are shown on the far right.

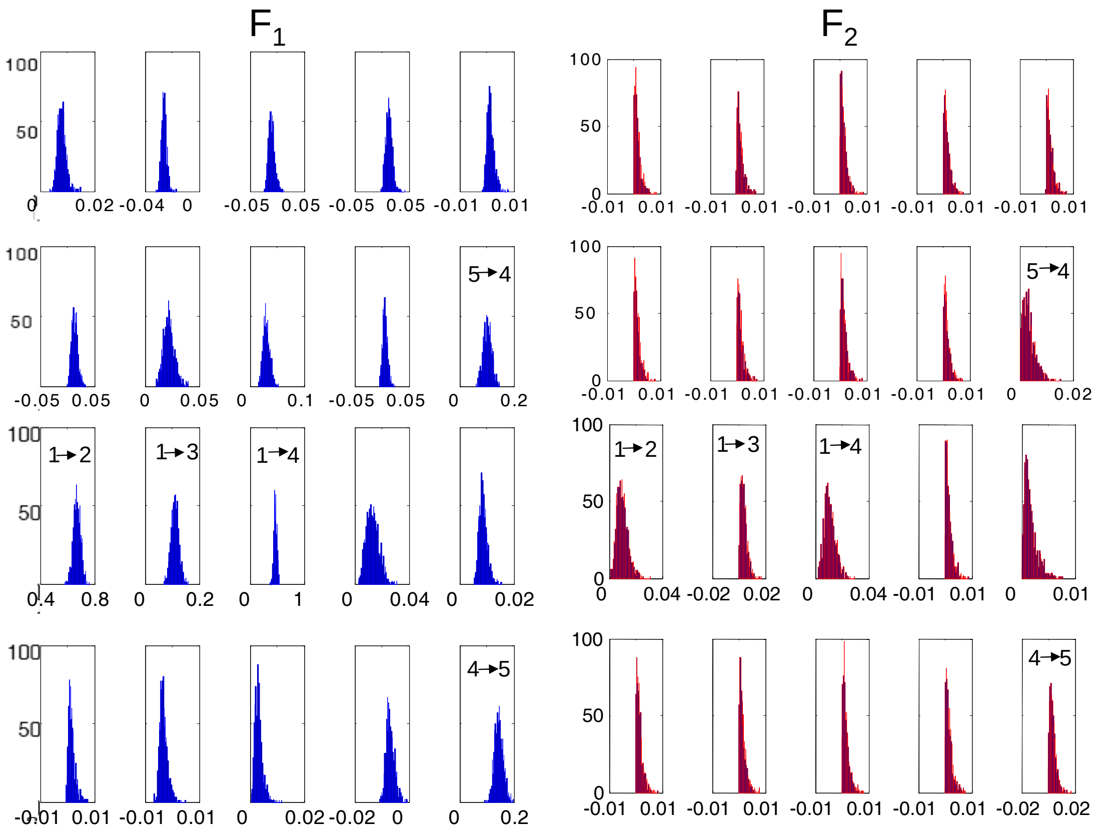

Figure 2: Histograms of F_1 and F_2 when $a_i = 0, b_i = 0, c_i = 0, i = 1, \cdots, 5$ in Example 1. Significant causalities are marked with links, i.e. the links $5 \to 4$, $1 \to 2$, $1 \to 3$, $1 \to 4$ and $4 \to 5$.

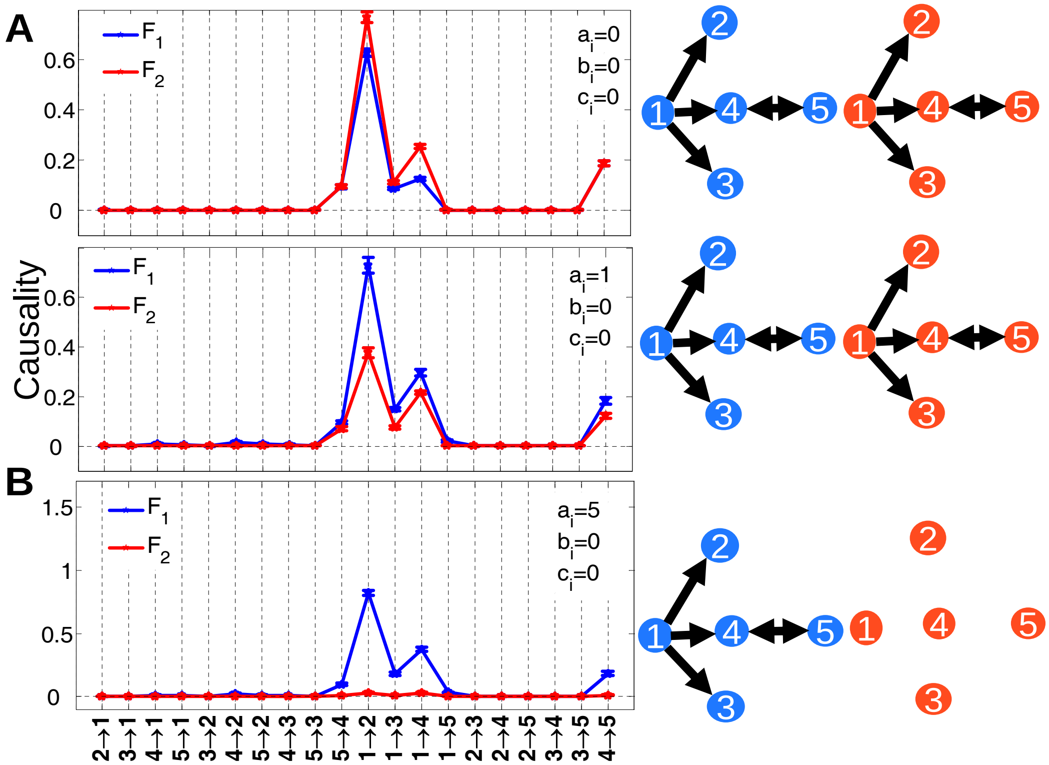

Figure 3: F_1, F_2 for different common exogenous inputs without latent variables (Example 1). (**A**) Left trace, F_1 (blue solid lines) and F_2 (red dotted lines) together with confidence intervals for all edges for $a_i = 0$ and $a_i = 1$. Right trace, the inferred causal relationships. A causal connection illustrated as part of the network if and only if the lower bound of the 95% confidence interval of the (partial, conditional) Granger causality is greater than zero. It is clearly seen that the correct causal relationships are recovered by both F_1 and F_2. (**B**) Left trace, F_1 (blue solid lines) and F_2 (red dotted lines) together with confidence intervals for all edges for $a_i = 5$; right trace, the inferred causal relationships. In this case F_2 does not identify any of the underlying causal connections.

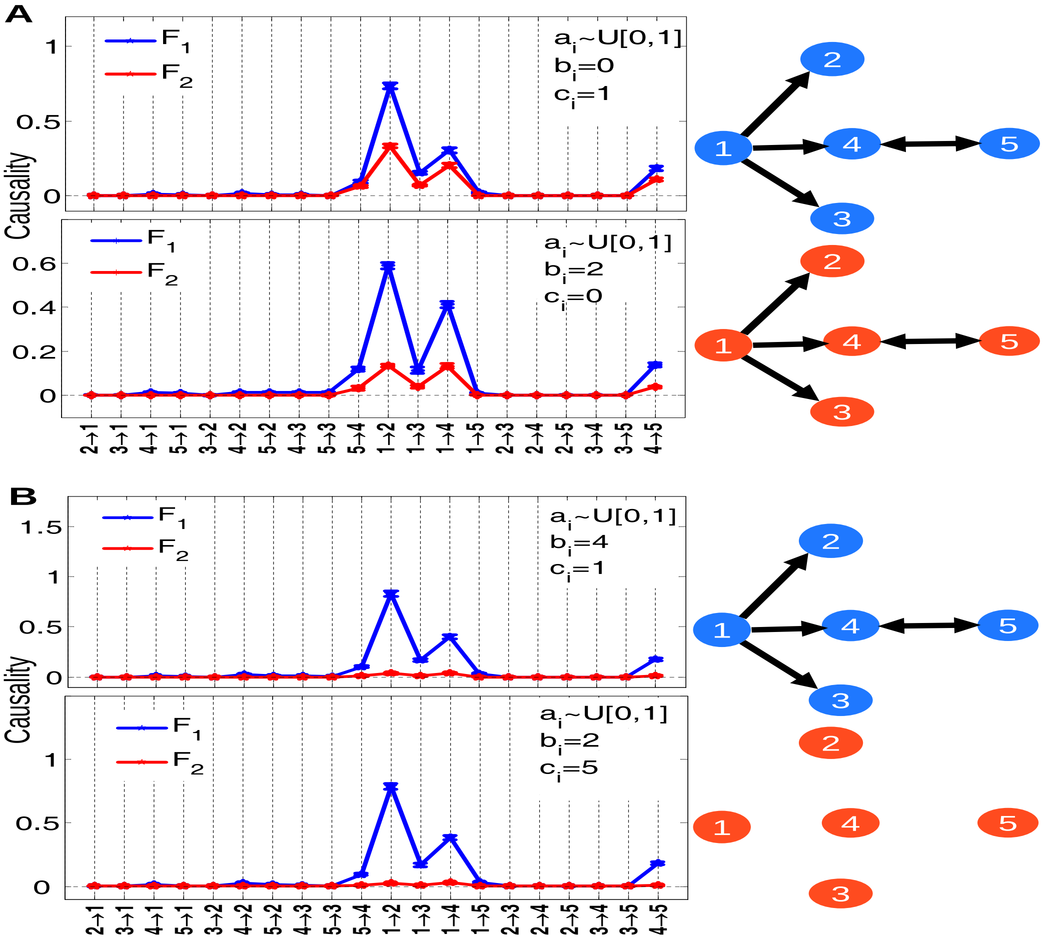

Figure 4: Results of example 1 when latent variables present. In these figures, $a_i \sim U[0,1], i = 1, \cdots, 5$.(**A**) $b_i = 0, c_i = 1$ and $b_i = 2, c_i = 0$ for all $i = 1, \cdots, 5$, both F_1 and F_2 are robust. (**B**) $b_i = 4, c_i = 1$ and $b_i = 2, c_i = 5$, in these cases, the causal relationships predicted from F_1 are unchanged, but in terms of F_2 no relationships are detected. A causal connection illustrated as part of the network if and only if the lower bound of the 95% confidence interval of the (partial, conditional) Granger causality is greater than zero.

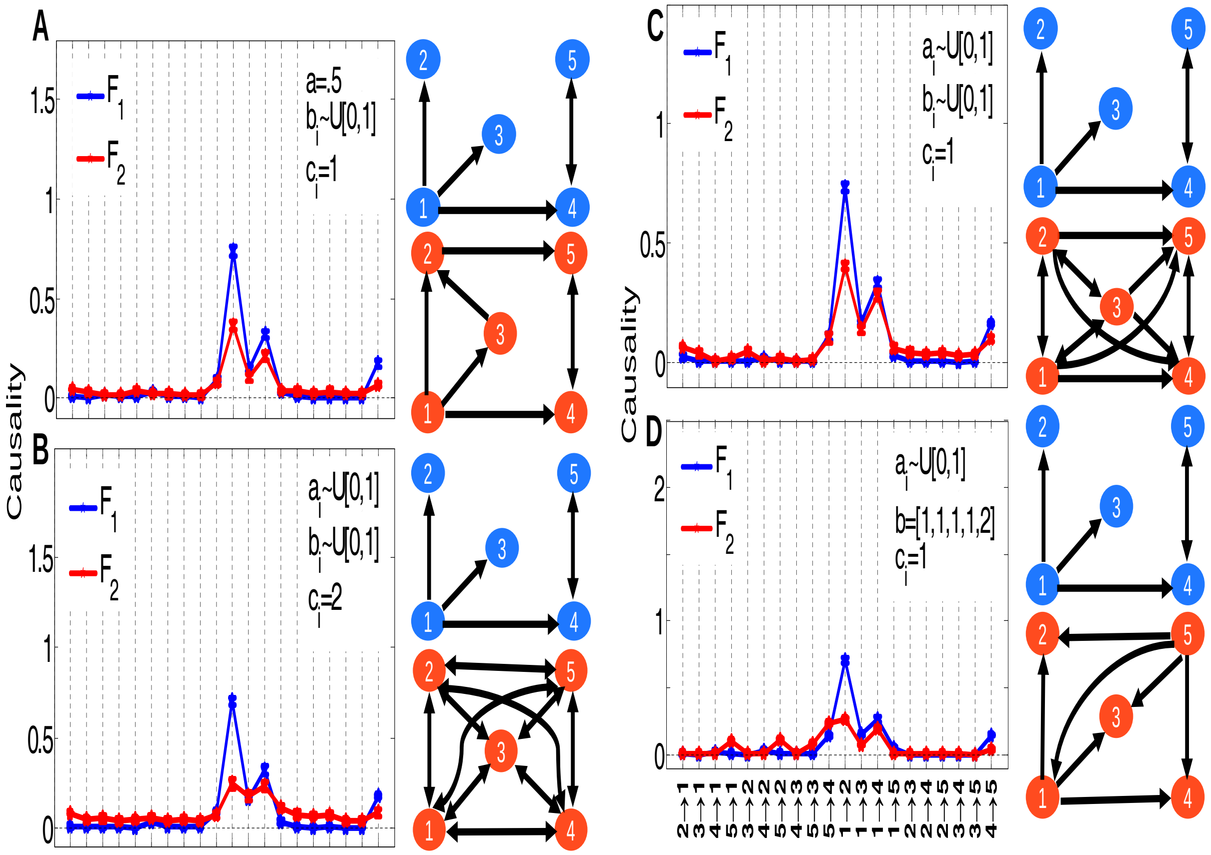

Figure 5: F_1 (blue solid lines) and F_2 (red dotted lines) and the corresponding inferred causal relationships, when the latent terms are randomly selected (example 1). F_1 is again robust while F_2 generates several false positives. A causal connection illustrated as part of the network if and only if the lower bound of the 95% confidence interval of the (partial, conditional) Granger causality is greater than zero.

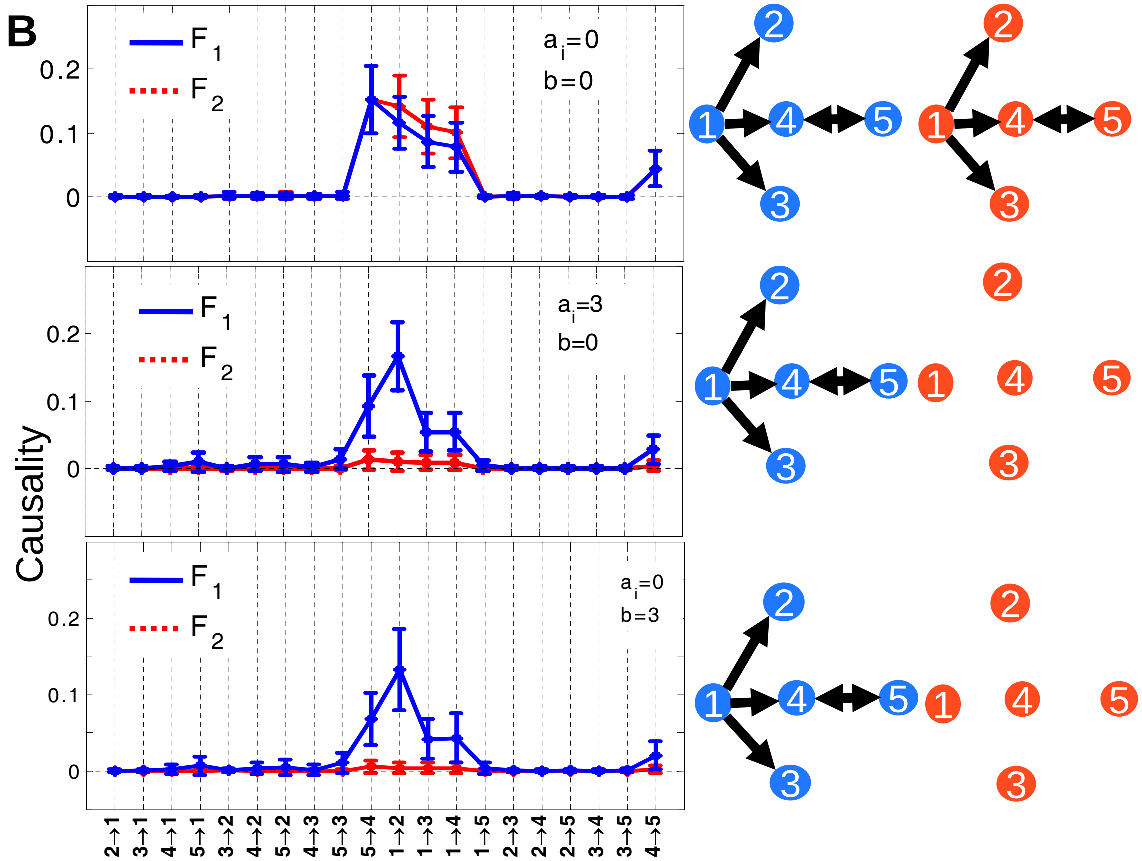

Figure 6: Data traces and predicted structures from the nonlinear model (Example 2). (**A**) Five time series used in our simulations. For visualization purposes, x_2, x_3, x_4, x_5 are shifted upward. (**B**) $a_i = 0, b = 0$, no exogenous input and latent variables are presented. In this case, both F_1 and F_2 predict the correct interactions. (**C**) $a_i = 3, b = 0$ and $a_i = 0, b = 3$, the predicted structures from F_1 are correct, but there is no interactions in terms of F_2. A causal connection illustrated as part of the network if and only if the lower bound of the 95% confidence interval of the (partial, conditional) Granger causality is greater than zero.

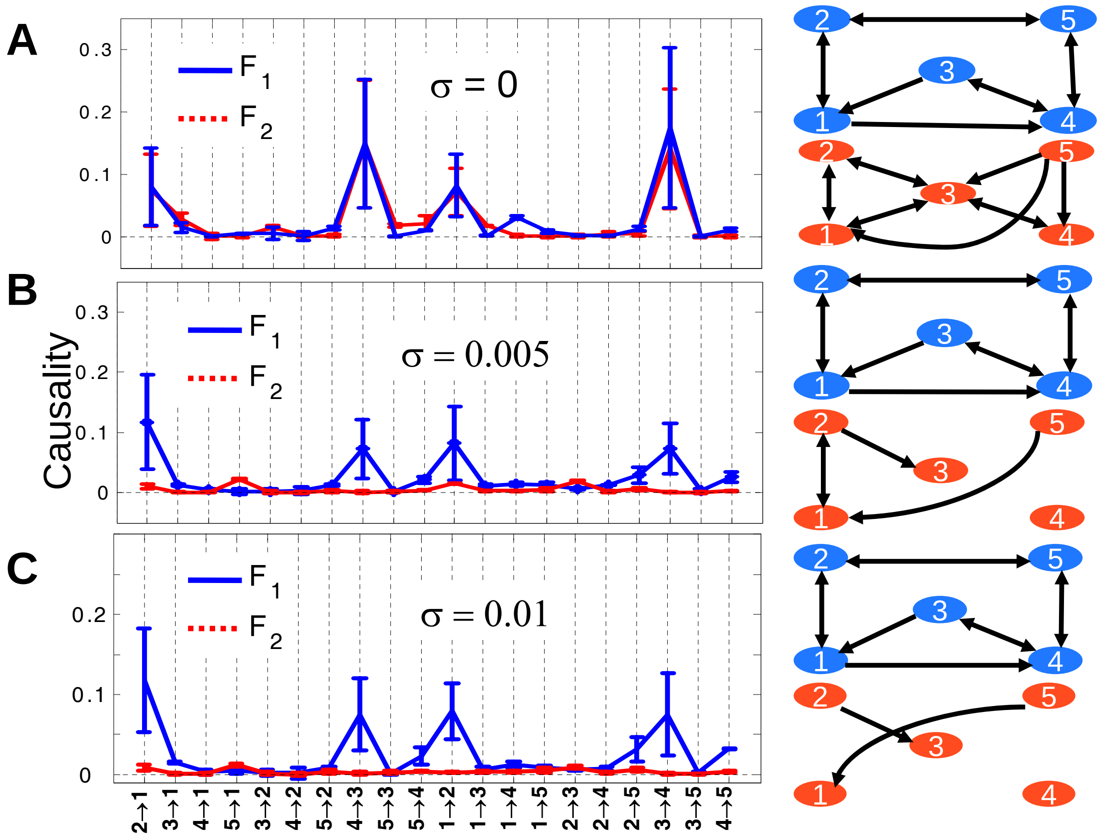

Figure 7: Results for experimental data. Exact values and confidence intervals are shown for F_1 (blue solid lines) and F_2 (red dotted lines) for $\sigma = 0$ (**A**), $\sigma = 0.005$ (**B**) and $\sigma = 0.01$ (**C**) respectively. The network is unchanged for F_1, but significantly changed for F_2 as a function of σ. Acausal connection illustrated as part of the network if and only if the lower bound of the 95% confidence interval of the (partial, conditional) Granger causality is greater than zero.
