# Supplementary material for: Uncovering Interactions in the Frequency Domain
Source: PLoS Comput Biol. 2008 May 30;4(5):e1000087. doi: 10.1371/journal.pcbi.1000087 (PMC2398781; doi:10.1371/journal.pcbi.1000087)
Supplement: Text S2 — A simple example in the frequency domain: dependence of the Granger causality on model parameters. (0.04 MB PDF) [file pcbi.1000087.s002.pdf]

5 Supplemental material I: A simple example

As an illustration of these definitions, we consider a simple system

$$\begin{cases} X_t = aX_{t-1} + bZ_{t-1} + \epsilon_{1t} \\ Z_t = cX_{t-1} + dZ_{t-1} + \epsilon_{2t} \end{cases} \tag{19}$$

with the covariance of the error terms

$$S = \begin{bmatrix} S_{11} & S_{12} \\ S_{21} & S_{22} \end{bmatrix}$$

Then we can rewrite the above model in terms of the lag operator

$$\begin{pmatrix} a_{11}(L) & a_{12}(L) \\ a_{21}(L) & a_{22}(L) \end{pmatrix} \begin{pmatrix} X_t \\ Z_t \end{pmatrix} = \begin{pmatrix} \epsilon_{1t} \\ \epsilon_{2t} \end{pmatrix} \tag{20}$$

Fourier transforming both sides of eq.(20) leads to

$$\begin{pmatrix} a_{11}(\lambda) & a_{12}(\lambda) \\ a_{21}(\lambda) & a_{22}(\lambda) \end{pmatrix} \begin{pmatrix} X(\lambda) \\ Z(\lambda) \end{pmatrix} = \begin{pmatrix} E_x(\lambda) \\ E_z(\lambda) \end{pmatrix} \tag{21}$$

where $a_{11}(\lambda) = 1 - ae^{-i\lambda}, a_{12}(\lambda) = -be^{-i\lambda}, a_{21}(\lambda) = -ce^{-i\lambda}, a_{22}(\lambda) = 1 - de^{-i\lambda}$

To drive the spectral decomposition of the time domain partial Granger causality, we multiply the matrix

$$P_1 = \begin{pmatrix} 1 & -S_{12}S_{22}^{-1} \\ \mathbf{0} & 1 \end{pmatrix} \tag{22}$$

to both sides of eq. (21). The normalized equations are represented as

$$\begin{pmatrix} \mathbf{D}_{11}(\lambda) & \mathbf{D}_{12}(\lambda) \\ \mathbf{D}_{21}(\lambda) & \mathbf{D}_{22}(\lambda) \end{pmatrix} \begin{pmatrix} X(\lambda) \\ Z(\lambda) \end{pmatrix} = \begin{pmatrix} X^*(\lambda) \\ Z^*(\lambda) \end{pmatrix} \tag{23}$$

with

$$\begin{aligned} D_{11}(\lambda) &= a_{11}(\lambda) - S_{12}S_{22}^{-1}a_{21}(\lambda) = 1 - (a - cS_{12}S_{22}^{-1})e^{-i\lambda} \\ D_{12}(\lambda) &= a_{12}(\lambda) - S_{12}S_{22}^{-1}a_{22}(\lambda) = -S_{12}S_{22}^{-1} - (b - dS_{12}S_{22}^{-1})e^{-i\lambda} \\ D_{21}(\lambda) &= a_{21}(\lambda) = -ce^{-i\lambda} \\ D_{22}(\lambda) &= a_{22}(\lambda) = 1 - de^{-i\lambda} \end{aligned} \tag{24}$$

and the determinant of matrix $\mathbf{D}$ is

$$\begin{aligned}\text{det}(D) &= D_{11}(\lambda)D_{22}(\lambda) - D_{12}(\lambda)D_{21}(\lambda) \\ &= 1 - (a - cS_{12}S_{22}^{-1})e^{-i\lambda} - de^{-i\lambda} + d(a - cS_{12}S_{22}^{-1})e^{-2i\lambda} \\ &\quad -cS_{12}S_{22}^{-1}e^{-i\lambda} - c(b - dS_{12}S_{22}^{-1})e^{-2i\lambda} \\ &= 1 - (a+d)e^{-i\lambda} + (ad - bc)e^{-2i\lambda}\end{aligned} \tag{25}$$

The elements of the transform function $\mathbf{G}$ are

$$\begin{aligned}G_{11}(\lambda) &= \frac{D_{22}(\lambda)}{\text{det}(D)} \\ &= \frac{1 - de^{-i\lambda}}{1 - (a+d)e^{-i\lambda} + (ad-bc)e^{-2i\lambda}} \\ &= \frac{(1 - de^{-i\lambda})}{M}[(1 - (a+d)\cos\lambda + (ad-bc)(\cos^2\lambda - \sin^2\lambda)) \\ &\quad -((a+d)\sin\lambda - 2(ad-bc)\sin\lambda\cos\lambda)i] \\ &= \frac{1 + d(a+d) - (a+2d)\cos\lambda + (\cos^2\lambda - \sin^2\lambda - d\cos^3\lambda - d\cos\lambda\sin^2\lambda)(ad-bc)}{M} \\ &\quad +i\frac{-(a + d(ad-bc))\sin\lambda + 2(ad-bc)\sin\lambda\cos\lambda}{M}\end{aligned}$$

$$\begin{aligned}G_{12}(\lambda) &= -\frac{D_{21}(\lambda)}{\text{det}(D)} \\ &= \frac{ce^{-i\lambda}}{1 - (a+d)e^{-i\lambda} + (ad-bc)e^{-2i\lambda}} \\ &= \frac{(ce^{-i\lambda})}{M}[(1 - (a+d)\cos\lambda + (ad-bc)(\cos^2\lambda - \sin^2\lambda) \\ &\quad -((a+d)\sin\lambda - 2(ad-bc)\sin\lambda\cos\lambda)i] \\ &= \frac{-c(a+d) + c(1 + ad - bc)\cos\lambda - i(1 - ad + bc)c\sin\lambda}{M}\end{aligned}$$

where $M = (1 - (a+d)\cos\lambda + (ad-bc)(\cos^2\lambda - \sin^2\lambda))^2 + ((a+d)\sin\lambda - 2(ad - bc)\sin\lambda\cos\lambda)^2$.

The spectra of the series $X(t)$ is

$$
\begin{aligned}
S_{xx}(\lambda) &= G_{11}(\lambda)(S_{11}-S_{12}S_{22}^{-1}S_{21})G'_{11}(\lambda)+G_{12}(\lambda)S_{22}G'_{12}(\lambda) \\
&= \frac{S_{11}-S_{12}S_{22}^{-1}S_{21}}{M^2}[(1+d(a+d)-(a+2d)\cos\lambda+ \\
&(\cos^2\lambda-\sin^2\lambda-d\cos^3\lambda-d\cos\lambda\sin^2\lambda)(ad-bc))^2 \\
&+((a+d(ad-bc))\sin\lambda+2(ad-bc)\sin\lambda\cos\lambda)^2] \\
&+\frac{S_{22}}{M^2}[(c(a+d)-c(1+ad-bc)\cos\lambda)^2+((1-ad+bc)c\sin\lambda)^2]
\end{aligned}
$$

The partial Granger causality from Y to X at the frequency λ is

$$
\begin{aligned}
f_{Y\to X|Z}(\lambda) &= \ln\frac{|S_{x^*x^*}(\lambda)|}{|Q_{xx}(\lambda)\hat{\Sigma}_{xx}Q'_{xx}(\lambda)|} \\
&= \ln\frac{S_{xx}}{G_{11}(\lambda)(S_{11}-S_{12}S_{22}^{-1}S_{21})G'_{11}(\lambda)} \\
&= \ln\left(1+\frac{G_{12}(\lambda)S_{22}G'_{12}(\lambda)}{G_{11}(\lambda)(S_{11}-S_{12}S_{22}^{-1}S_{21})G'_{11}(\lambda)}\right)
\end{aligned}
$$

Our calculations above indicate that even for a simple model, the relationship between the model coefficients and its partial Granger causality in the frequency domain is not simple at all, although the relationship in the time domain is almost trivial. The dependence of the partial Granger causality in the frequency domain on its coefficients in the time domain is highly nonlinear.
